# Convergent resistance to GABA receptor neurotoxins through plant–insect coevolution

**DOI:** 10.1038/s41559-023-02127-4

**Published:** 2023-07-17

**Authors:** Lei Guo, Xiaomu Qiao, Diler Haji, Tianhao Zhou, Zhihan Liu, Noah K. Whiteman, Jia Huang

**Affiliations:** 1 Ministry of Agriculture Key Laboratory of Molecular Biology of Crop Pathogens and Insects, Institute of Insect Sciences, Zhejiang University, Hangzhou, China; 2 Xianghu Laboratory, Hangzhou, China; 3 Department of Integrative Biology, University of California, Berkeley, CA USA; 4 Department of Molecular and Cell Biology, University of California, Berkeley, CA USA

**Keywords:** Coevolution, Functional genomics

## Abstract

The molecular mechanisms of coevolution between plants and insects remain elusive. GABA receptors are targets of many neurotoxic terpenoids, which represent the most diverse array of natural products known. Over deep evolutionary time, as plant terpene synthases diversified in plants, so did plant terpenoid defence repertoires. Here we show that herbivorous insects and their predators evolved convergent amino acid changing substitutions in duplicated copies of the *Resistance to dieldrin* (*Rdl*) gene that encodes the GABA receptor, and that the evolution of duplicated *Rdl* and terpenoid-resistant GABA receptors is associated with the diversification of moths and butterflies. These same substitutions also evolved in pests exposed to synthetic insecticides that target the GABA receptor. We used in vivo genome editing in *Drosophila melanogaster* to evaluate the fitness effects of each putative resistance mutation and found that pleiotropy both facilitates and constrains the evolution of GABA receptor resistance. The same genetic changes that confer resistance to terpenoids across 300 Myr of insect evolution have re-evolved in response to synthetic analogues over one human lifespan.

## Abstract (web summary)

Antagonistic coevolution of plants and insects is hypothesized to promote biological diversity. This study shows convergent evolution of a gene that encodes the GABA receptor, a target of plant toxins, in herbivorous insects and their predators. It further demonstrates that the evolution of resistant GABA receptors is associated with insect diversification.

## Main

Herbivorous insects and their host plants represent the most abundant and diverse forms of macroscopic life on Earth. Their extraordinary contemporary diversity is hypothesized to have arisen from antagonistic coevolution between the two wherein defence and counter-defence reciprocally drove diversification^1,2^. The escape-and-radiate model^3^ of coevolution provides one historical scenario for explaining patterns of evolutionary diversification in plants and herbivores. It proposes that novel plant chemical defences evolved in response to herbivore-induced selection, promoting an adaptive radiation in a focal plant lineage. This in turn drove the evolution of new insect counter-defence mechanisms and an associated radiation in a specialized herbivore lineage that colonizes these plants. For example, the ancestral Brassicales (mustards and relatives) produced glucosinolates as a novel chemical defence, the ‘mustard oil bomb’, through the duplication and neofunctionalization of genes in the cyanogenic glucoside pathway. This was followed by the evolution of a novel detoxification mechanism in ancestral Pierinae butterfly larvae that permitted a host plant shift from Fabales to Brassicales and resulted in the adaptive radiation of the herbivores^3,4^. While consistent with the escape-and-radiate model, our general understanding of the genetic and molecular bases of plant–herbivore coevolution and their potential role in adaptive radiations^5,6^ remains limited.

Terpenoids are the most chemically and structurally diverse compounds known in plants, representing approximately 60% of all known natural products^7^. The effects of terpenoids on herbivores vary from beneficial to lethal, but many terpenoids are repellent and neurotoxic^8^. For example, naturally occurring pyrethrins are sesquiterpenoids produced by chrysanthemums, the powder of which has been used as a botanical insecticide for thousands of years^9^. These terpenoids impair neuronal function by binding to voltage-gated sodium channels. Picrotoxin, another natural sesquiterpenoid, acts as a non-competitive antagonist (NCA) on GABA_A_ receptors. These pentameric ligand-gated ion channels conduct bicarbonate and chloride ions in the central nervous system of vertebrates and invertebrates. In addition, a wide range of terpenoids act as NCAs or positive allosteric modulators (PAMs) at GABA_A_ receptors, including the monoterpenoids α-thujone and thymol, the sesquiterpenoids bilobalide and ginkgolides, and the diterpenoids isopimaric acid and miltirone^10–12^. Thus, a diversity of evolutionary strategies exists, through which herbivorous insects have specialized on defensive terpenoid-containing tissues from across the entire diversity of plants, but such counter-defence mechanisms remain poorly known.

Unlike their mammalian homologues in which different classes of GABA_A_ subunits form a diverse family of hetero-oligomers, the structure and assembly of insect ionotropic GABA receptors have not been fully elucidated^13^. The insect GABA receptor studied most intensively is encoded by *Rdl*, which was initially characterized in *Drosophila melanogaster* through a genetic screen for mutations associated with dieldrin resistance^14^. Dieldrin is a synthetic organochloride (cyclodiene) insecticide first produced in the USA in 1948. The *Rdl*-encoded subunits form functional homo-oligomeric chloride channels, the pharmacology of which is similar to that of native GABA receptors in insect nervous systems^15,16^. Other ionotropic receptor subunits, such as GRD (GABA and glycine receptor-like subunit from *Drosophila*) and LCCH3 (ligand-gated chloride channel homologue 3), form GABA-gated cation channels when heterologously expressed^17^. Furthermore, *Rdl* orthologues have been identified in the genomes of many other insect species, most of which contain one copy. However, genomes of three moth^18–20^ and two aphid^21,22^ lineages each contain two copies of *Rdl*.

Interestingly, a point mutation at position 302 that resulted in a single non-synonymous replacement of an alanine with serine (A302S or A2ʹS, index number for the M2 membrane-spanning region) in the *Rdl* gene of some wild strains of *D. melanogaster* confers resistance not only to NCA insecticides such as dieldrin and fipronil^11,16^ but also to diverse plant-produced natural terpenoids, including picrotoxin, α-thujone, bilobalide and ginkgolides^23,24^. This cross-resistance pattern led us to hypothesize that *Rdl* duplications and parallel amino acid substitutions at position 2ʹ in RDL may be a window into an uncharacterized resistance mechanism that evolved over deep time against neurotoxic terpenoids via target site insensitivity at insect GABA receptors. Here we addressed this hypothesis using molecular evolution, structure–function, diversification rate and precision genome editing approaches to study the effects of the resistance mutations in vivo.

## Molecular evolution of *Rdl* in insects

**Extended Data Fig. 1:**
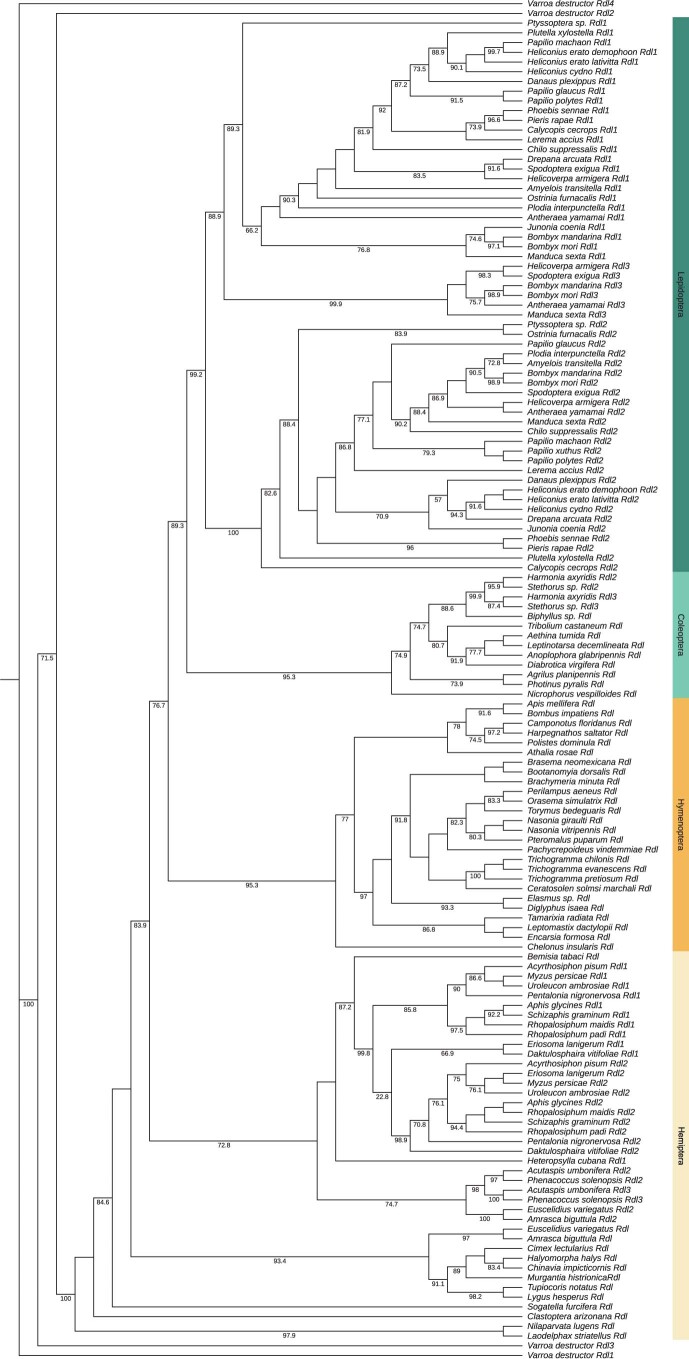
Phylogenetic relationships of RDL in insects. Maximum likelihood based phylogenetic tree of RDL from four orders: Hemiptera, Hymenoptera, Coleoptera, and Lepidoptera. Protein sequence alignment was performed using MUSCLE in MEGA X. The best-fit model was VT + I + G4 estimated using ModelFinder, and a maximum likelihood (ML) tree inference was subsequently conducted in iqtree with default parameters. The four RDL subunit sequences of the mite *Varroa destructor* were used as outgroups. The values on the branches represent bootstrap support. The deep divergence of *Rdl* duplications in three orders suggests duplications have occurred before the diversification of their respective lineages. For a complete list of taxa surveyed see Supplementary Table 1.

**Extended Data Fig. 2:**
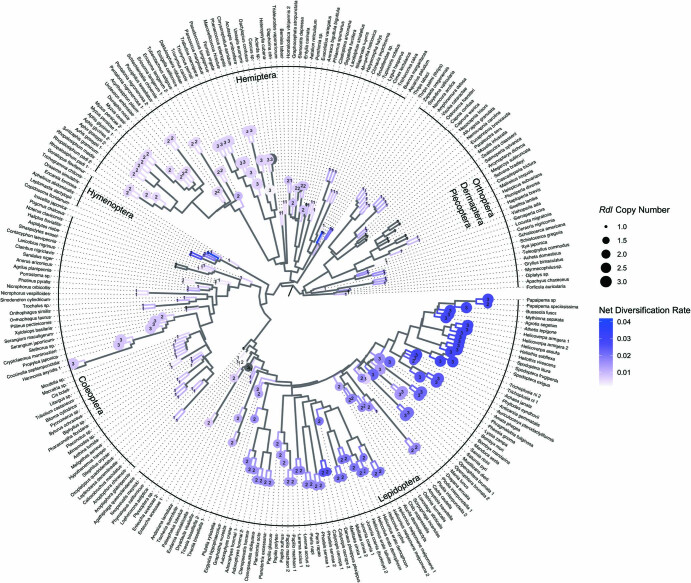
Species-level analysis of the relationship between *Rdl* duplications and net diversification rate. ASTRAL species tree of all insect species analyzed. The species tree was constructed using gene trees of 1123 single-copy orthologs extracted using the BUSCO pipeline from published genome and transcriptome assemblies. Gene trees were inferred from amino acid alignments using maximum-likelihood estimation in IQ-Tree. Net diversification rates were calculated using the extant species richness of the associated family of each taxon, and the stem age (Ma) of that family taken from previously published time-calibrated phylogenies. *Rdl* copy number is provided within tip points and was determined by manual inspection of each genome and transcriptome assembly.

After mining existing databases, we aligned *Rdl* sequences from 22 orders and 171 insect families together with species with known pesticide resistance evolution, to directly assess duplication and candidate site evolution. The *Rdl* gene is present as a single copy in most lineages (22 orders and 132 families). However, 2–3 copies are found across genomes of 52 aphid (Aphidomorpha: Phylloxeroidea and Aphidoidea), scale insect (Coccoidea), treehopper and leafhopper (Membracoidea), ladybird (Coccinellidae), and moth and butterfly (Lepidoptera) species. After the initial duplication events, one paralogue retaining the ancestral *Rdl* sequences without resistance substitutions was lost in both scale insects and ladybirds (Supplementary Table 1). In addition, in 79 transcriptomes of species from the above-mentioned families, we observed the same gene duplications and losses in 24 species; however, one or two paralogues were not found in 55 species (Supplementary Table 1). The transcriptomes from these species may be incomplete due to sequencing depth or sampling bias, because *Rdl* is primarily expressed in the central nervous system. Despite this uncertainty, at least eight independent duplications and losses of *Rdl* preceded the diversification of these insect lineages (Extended Data Figs. 1 and 2, and Supplementary Table 2).

**Fig. 1:**
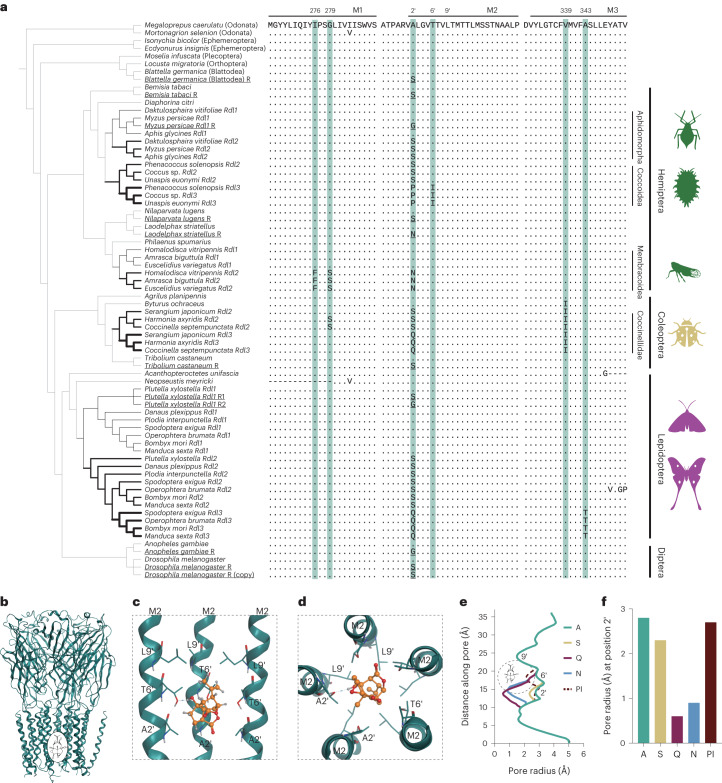
Molecular evolution of *Rdl* in insects. **a**, Maximum-likelihood phylogeny of RDL using an amino acid alignment of translated *Rdl* genes from resistant and non-resistant insect species, including *Rdl* duplications and amino acid substitutions (see Supplementary Tables 1 and 3 for all examined species). Species include those evolving resistance substitutions in the context of coevolutionary adaptation to host plants over deep time (not underlined) and those that have evolved resistance mutations on contemporary timescales in response to insecticides (underlined, *n* = 9 species). The first set of duplicated copies are named *Rdl2* (for example, 2ʹS and 2ʹN) and the second set of duplicated copies are named *Rdl3* (for example, 2ʹQ and 2ʹP). Black thick branches represent inferred duplications of *Rdl* (medium thick *Rdl2* and thick *Rdl3*). The amino acid substitutions of six sites (I276, G279, A2ʹ, T6ʹ, V339 and A343) are highlighted in blue. Amino acid substitutions at positions I276 and G279 were found in the *Rdl2* copies in treehopper and leafhopper species; the amino acid A2ʹ was replaced in all the species containing duplicated *Rdl* copies and a subset of species that are resistant to synthetic insecticides; the amino acid G279 was replaced in some ladybird lineages; the amino acid V339 was replaced in *Byturus ochraceus* and ladybirds; the amino acid of A343 was replaced only in the *Rdl3* copies of moths. Aphidomorpha, aphids (Phylloxeroidea and Aphidoidea); Coccoidea, scale insects; Coccinellidae, ladybirds; and Membracoidea, treehoppers and leafhoppers. **b**–**d**, Model of the picrotoxin-bound *D. melanogaster* RDL (insect GABA receptor) homo-multimer. View of the binding pocket from the parallel to the membrane plane (**b**), and side-on (**c**) and down-top (**d**) views of the picrotoxin-bound channel pore. Picrotoxinin, the main active ingredient of picrotoxin, is shown in ball-and-stick, side chains of amino acid residues are shown as sticks, dashed lines indicate hydrogen bonds, and membrane-spanning segments are indicated by dark-green ribbons. **e**,**f**, Plot of the pore radii (**e**) and pore radius at position 2ʹ (**f**) in wild-type and mutant RDL models. A, wild-type; S, A2ʹS; Q, A2ʹQ; N, A2ʹN; and PI, A2ʹP-T6ʹI. The dashed circle indicates the binding pocket of picrotoxin.

We found that position 2ʹ experienced amino acid substitutions in all duplicated *Rdl* copies. 2ʹS was identified in *Rdl2* copies of all species except treehoppers and leafhoppers, in which a unique substitution 2ʹN was identified, and 2ʹQ was identified in *Rdl3* copies of ladybirds and moths. There are double amino acid substitutions (2ʹP-6ʹI) in *Rdl3* copies of scale insects (Fig. 1a).

These substitutions are exclusively associated with ancient duplication events of *Rdl*. Remarkably, segregating mutations at position 2ʹ, such as A2ʹS, A2ʹN and A2ʹG, have also been found in contemporary populations of more than 24 species from 6 arthropod orders in the past 30 yr^25^ (Fig. 1a and Supplementary Table 3). These *Rdl* mutations confer resistance to synthetic cyclodiene and phenylpyrazole insecticides via target site insensitivity^16,26^. In addition, four positions in M1 (I276 and G279) and M3 (V339 and A343) have also experienced protein coding mutations in *Rdl* copies of different lineages, including treehoppers and leafhoppers, the fruitworm beetle *Byturus ochraceus*, ladybirds and moths (Fig. 1a).

The binding site for NCAs, including picrotoxin and the synthetic cyclodiene and phenylpyrazole insecticides, is formed by the pore-lining 2ʹ, 6ʹ and 9ʹ residues in both insect and mammalian GABA receptors^11,16,26,27^ (Fig. 1b–d). Point mutations at these sites can reduce or abolish the potentiation effect of PAMs such as benzodiazepines, general anaesthetics and thymol^28–30^. However, the binding mode of benzodiazepines, such as diazepam and alprazolam, is in the extracellular domain and M3–M1 interfaces, and general anaesthetics such as barbiturates and propofol target the M3–M1 interfaces^27,31^, suggesting that the substitutions in the M1 and M3 regions of *Rdl* copies may be involved in PAM binding. Notably, 72 terpenoids with diverse structures distributed across gymnosperms and angiosperms act on GABA receptors as NCAs or PAMs (Supplementary Tables 4 and 5) and many others probably share the same modes of action.

**Extended Data Fig. 3:**
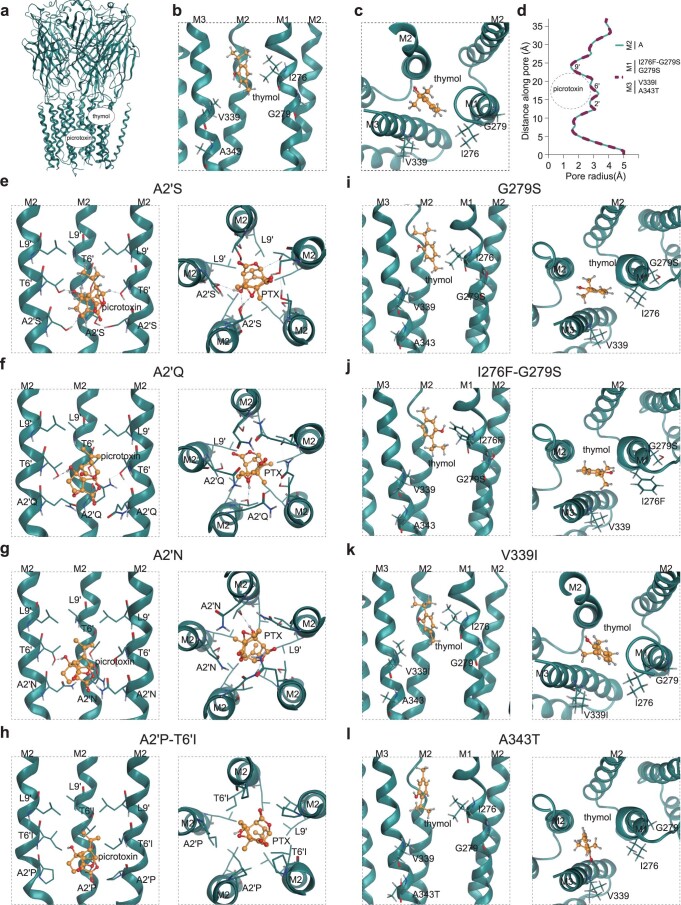
View of the binding sites in the picrotoxin-bound RDL structures for each of the 2′ substitutions with the representative top poses (related to Fig. 1). (**a** to **c**) Model of the thymol (**a–c**)-bound *Drosophila melanogaster* RDL homo-pentamer. View of the binding pocket from the parallel to the membrane plane (**a**). Side-on (**b**) and down-top (**c**) views of the picrotoxin and thymol-bound channel pore. (**d**) Plot of the pore radii in wild-type and mutant RDL models. A: wild-type. (**e–l**) The docking scores of the picrotoxin poses (top) are −7.09, −6.18, −7.73, and −7.18 in the structures of A2′S (**e**), A2′Q (**f**), A2′N (**g**), and A2′P-T6′I (**h**), respectively. The docking scores of the thymol poses (top) are −5.02, −5.08, −5.25, and −5.12 in the structures of G279S (**i**), I276F-G279S (**j**), V339I (**k**), and A343T (**l**), respectively. Picrotoxinin, the main active ingredient of picrotoxin, and thymol are shown in ball-and-stick, amino acid side chains are shown as sticks, and the dashed lines indicate hydrogen bonds.

We hypothesized that the ancient amino acid substitutions at these positions may confer resistance to host defensive terpenoids in the salient herbivorous lineages. To address this, we generated models containing amino acid replacements docked with picrotoxin and thymol using the crystal structure of the picrotoxin and propofol-bound human GABA_A_ receptor^27,31^ (Fig. 1b and Extended Data Fig. 3a). Our docking simulations suggest that the 2ʹ, 6ʹ and 9ʹ M2 residues are involved in picrotoxin binding (Fig. 1c,d), consistent with mutagenesis, electrophysiology and structural pharmacology results^16,26,27^. Picrotoxin forms hydrogen bonds with T6ʹ and hydrophobic interactions with A2ʹ, T6ʹ and L9ʹ. In M1 and M3 regions, molecular docking shows that the I276 can bind thymol and other positions near the thymol-binding pocket (Extended Data Fig. 3b,c). The A2ʹS mutation was identified in populations of many cyclodiene-resistant insect species^25^ and may result in steric hindrance of NCAs in the channel pore^16^(2.3 Å; Fig. 1e,f and Extended Data Fig. 3e) compared to the wild-type RDL (2.8 Å; Fig. 1e,f). The 2ʹQ and 2ʹN substitutions further reduced the pore radius to 0.6 Å and 0.9 Å, respectively, causing more steric hindrance (Fig. 1e,f and Extended Data Fig. 3f,g). However, the double substitution 2ʹP-6ʹI decreased the channel diameter at position 6ʹ (Fig. 1e,f and Extended Data Fig. 3h). Notably, the substitutions in M1 and M3 did not affect the channel-pore radius (Extended Data Fig. 3d,i–l). These results indicate that the substitutions could prevent the binding of the receptor with NCA and PAM terpenoids and may reduce the potentiation effect of PAM terpenoids. Overall, duplications and amino acid substitutions in *Rdl* may contribute to adaptation to insecticides and neurotoxic terpenoids.

## Evolution of *Rdl* is associated with herbivore host shifts and diversification

**Fig. 2:**
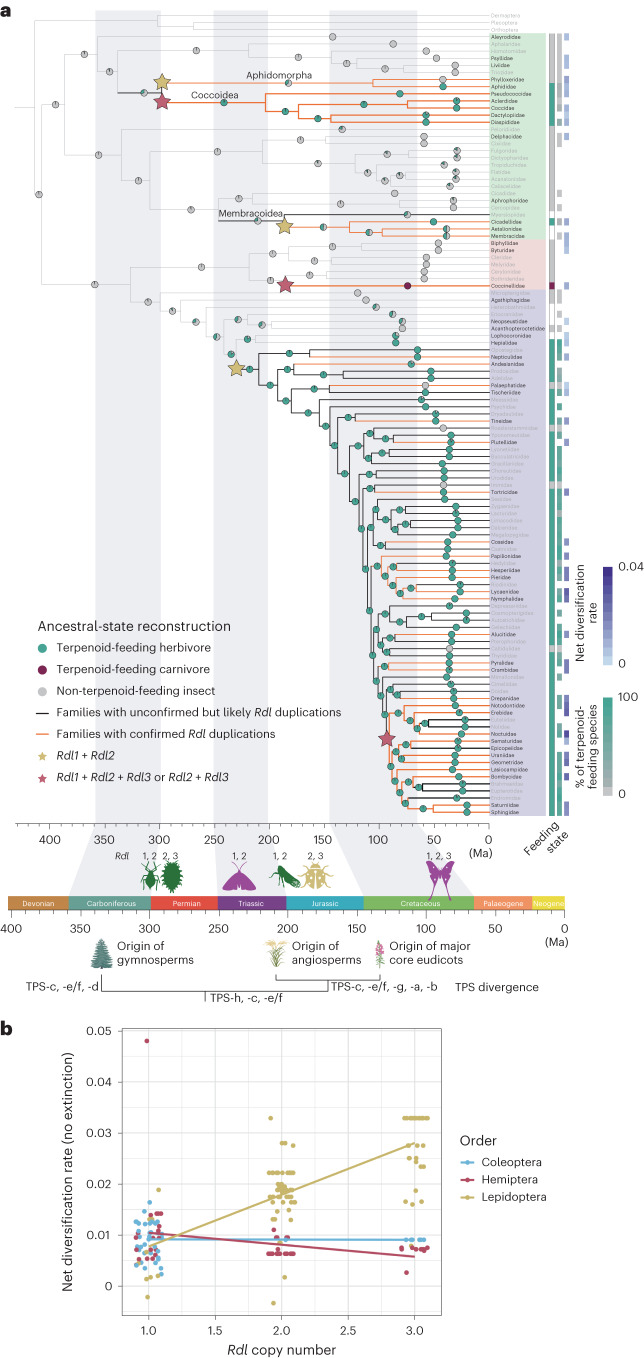
Evolution of *Rdl* is associated with herbivore host shifts and diversification. **a**, Family-level ancestral-state reconstructions of feeding states estimated from the character states of extant species: Green, terpenoid-feeding herbivore on gymnosperms (in Hemiptera) and angiosperms, which produce diverse terpenoids targeting insect GABA receptors; red, terpenoid-feeding carnivore of scale insects and aphids; grey, non-terpenoid-feeding insect; and white, unknown. Maximum-likelihood reconstructions are shown as nodal pie graphs. The orange and black thick branches represent inferred duplications of *Rdl* (medium thick *Rdl2* and thick *Rdl3*). Family names in black indicate that the *Rdl* sequence from at least one species in the family lineage is known. Green, pink, and purple background colours mark Hemiptera, Coleoptera and Lepidoptera, respectively. The heat map shows the percentage of terpenoid-feeding species (Supplementary Tables 6 and 7) and net diversification rate. The timeline relates the convergent evolution of *Rdl* duplications in insect taxa to the origin of gymnosperms and angiosperms, and the subsequent diversification of each. The evolution of terpene synthase genes (TPS) in plants is taken from ref. ^34^. The tree was rooted with sequences from Dermaptera (*Diplatys* sp.), Plecoptera (*Moselia infuscata*), and Orthoptera (*Myrmecophilus* sp.) (Supplementary Table 1). **b**, Relationship between *Rdl* copy number and net diversification rate across all insects surveyed for *Rdl* copy number variation and resistance evolution.

The most ancient *Rdl* duplication events probably arose ~298 million years ago (Ma) in the two common ancestors of aphids and scale insects, respectively (Fig. 2a). In the ancestral lineage of scale insects, two rounds of gene duplications and the loss of the ancestral version of the gene occurred. The *Rdl* gene in the ladybird lineage, which feed primarily on aphids and scale insects, experienced a parallel set of duplications and losses with respect to its prey ~186 Ma. The sister group to the rest of the ladybirds, the subfamily Sticholotidinae, carries two paralogues and has also lost the ancestral version of *Rdl* (Supplementary Table 1). The duplications found in treehoppers and leafhoppers appeared around the same time ~187 Ma or earlier since the *Rdl* sequence was not found in members of the sister family Myerslopiidae. Notably, the duplications that generated the *Rdl2* copy are older than the duplications that generated the *Rdl3* copy observed in the lepidopteran lineages (~230 Ma and ~94 Ma, respectively) (Fig. 2a). Thus, these results suggest that most duplication events occurred near the origin of angiosperms^32^, and only duplications that generated *Rdl3* in moths occurred after the origin of core eudicots. The relative sequential timing of key *Rdl* duplication events and subsequent terpenoid-resistance substitutions may dovetail with key events in the evolution of plants.

Accordingly, we investigated in more detail the potential for coevolution between insect and plant lineages in the context of the evolution of terpenoid defences and *Rdl* evolution. In plants, terpene synthases are enzymes necessary for the synthesis of terpenoids. The genes encoding terpene synthases originated in non-seed plants ~450 Ma and subsequently diversified in gymnosperms and angiosperms, particularly core eudicots, which together account for the extreme diversity of terpenoid chemicals in plants^33,34^.

**Extended Data Fig. 4:**
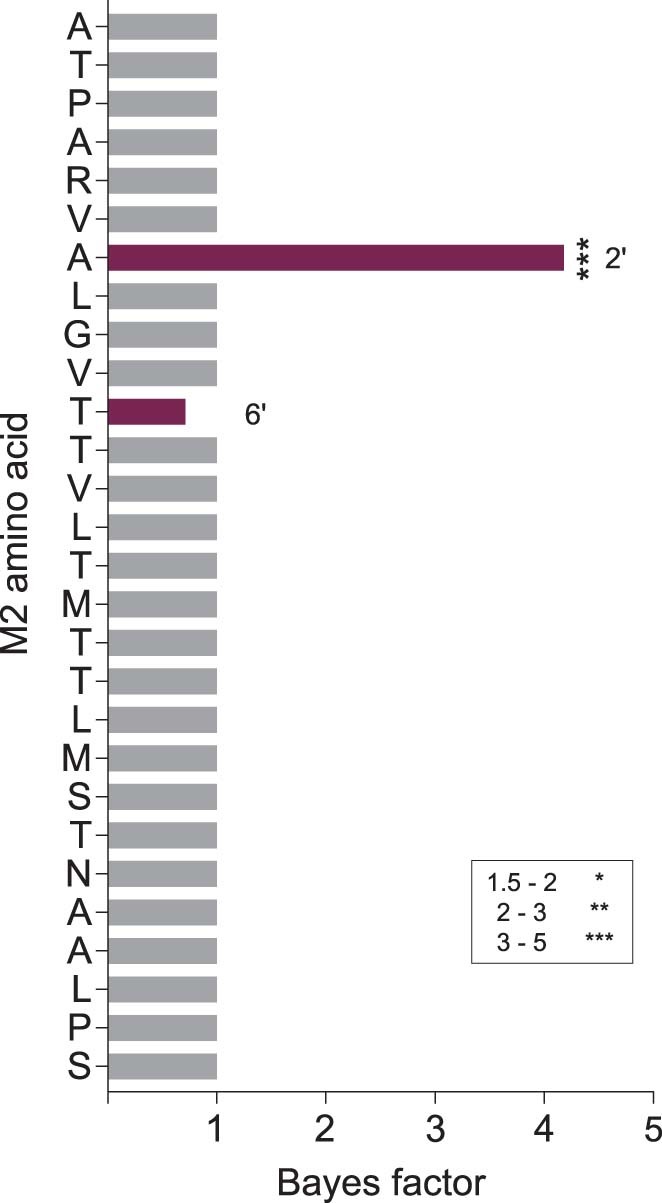
TraitRateProp analyses show the strong association between M2 2′ residues of RDL and feeding states in insects. A rooted ultrametric phylogenetic tree, multiple alignments of RDL, and trait states from three orders (Hemiptera, Coleoptera, and Lepidoptera) were used, and see more information in method details.

Many gymnosperm- and angiosperm-specific terpenoids serve as chemical defences against herbivores; for example, bilobalide and ginkgolides in gymnosperms, and picrotoxin and anisatin in angiosperms^11^. We observed that the lineages of aphids and scale insects feed far more extensively on gymnosperms that produce terpenoids targeting GABA receptors compared with related lineages. Although the hosts of most treehopper and leafhopper families are uncertain, species from the Cicadellidae also feed on gymnosperms that produce terpenoids targeting GABA receptors, while species from other related clades do not (Fig. 2a and Supplementary Table 6). In tandem, lepidopteran species with duplications of *Rdl* diversified on host plants that produce even more diverse terpenoids targeting GABA receptors (Fig. 2a and Supplementary Table 7). Previous studies have indicated that host plant chemistry played a significant role in the evolution of host shifts in herbivorous insects over deep time^3,35^. Our maximum-likelihood ancestral-state reconstructions of host preference suggest such successive host shifts from early non-vascular land plants (bryophytes), to gymnosperms and then to angiosperms over time. Each of these host plant shifts is coincident with the observed duplications and salient amino acid substitutions of *Rdl* (Fig. 2a). Furthermore, we found that the specific substitutions at the 2ʹ residues were strongly associated with host shifts (Extended Data Fig. 4).

We then studied the potential macroevolutionary consequences of the *Rdl* duplications and convergent amino acid substitutions for the insects in which they evolved. We first applied sister-clade analysis to test whether ancestral shifts in *Rdl* copy number are associated with shifts in diversification rate given extant species richness. We found a significant increase in diversification rate using six sister-taxon pairs associated with transitions in *Rdl* copy number (Table 1 and Supplementary Table 8).

**Table 1:** Sister-clade analysis results

Species pair removed from sister-clade analysis	Chi-squared	d.f.	*P* value
None	90.03757	1	2.3368 × 10^−21^
Aphidamorpha/Coccoidea: Psylloidea	43.84667	1	3.551349 × 10^−11^
Coccoidea: Aphidamorpha	35.41264	1	2.667503 × 10^−09^
Membracoidea: Cicadoidea/Cercopoidea	−8.580884	1	1
Coccinellidae: Cerylonidae/Bothrideridae	−8.581524	1	1
Heteroneura: Lophocoronoids/Hepialoids	−10.81037	1	1
Noctuoids/Lasiocampoids/Bombycoids: Drepanoids	−10.74069	1	1

We performed a full sister-clade analysis using all species pairs (no species pair was removed from sister-clade analysis). We also performed additional sister-clade analyses by removing one pair of sister clades to test for the contribution of any one pair on the full analysis (see Methods for more information). *P* values represent a one-sided test and were calculated using the ‘richness.yule.test’ function in the R package ape.

To complement the sister-clade analysis, we then estimated net diversification rates across all lineages in which we were able to curate *Rdl* sequences and for subsets of lineages representing the major insect orders with *Rdl* non-pesticide resistance evolution (Coleoptera, Hemiptera and Lepidoptera) under different extinction rate scenarios as in ref. ^36^ (0% extinction, 50% extinction relative to speciation rate, 90% extinction relative to speciation rate) (Fig. 2 and Extended Data Fig. 2). For the full dataset, we found that the *Rdl* copy number increased the net diversification rate for each extinction scenario (***P < 0.001). However, this effect was primarily driven by the Lepidoptera (***P < 0.0001), while there was no effect in the Coleoptera and a slightly negative effect on the diversification rate in the Hemiptera (*P < 0.05) (Fig. 2b and Supplementary Table 9).

We further tested the relationship between *Rdl* copy number and net diversification rate using a phylogenetic generalized least squares method to account for phylogenetic relatedness among the taxa studied. The *Rdl* copy number was positively correlated with the net diversification rate under all extinction scenarios (*P* < 0.004) and explained ~1–3% of the variation across all insect orders sampled after accounting for phylogenetic non-independence. This pattern was primarily driven by the Lepidoptera (*P* < 0.001) in which 10–14% of the variation in net diversification rates could be explained by the *Rdl* copy number alone. The effect of the *Rdl* copy number was not significant after accounting for phylogeny in the Hemiptera- or Coleoptera-specific datasets (*P* > 0.1). These results were recapitulated under both low and high lambda transformations (Supplementary Tables 10 and 11). Overall, our results imply that the evolutionary genetics changes in *Rdl* arose as a counter-defence mechanism that spurred adaptive radiations in Lepidoptera, which is among the most diverse insect orders. The increased diversification rate in Lepidoptera may have occurred through host plant shifts that enabled avoidance of the terpenoid toxins concurrently diversifying through terpene synthase gene duplications in plants during the Mesozoic (Fig. 2).

Many herbivores take up and store host plant toxins in defence against their own natural enemies^37^. However, it is not well understood how predators of insects have evolved the capacity to resist or tolerate plant defence metabolites sequestered within their prey^38^. Here we found that both scale insect prey and ladybird predators underwent a suite of parallel molecular evolutionary changes in sequential order: (1) duplication of *Rdl*; (2) nucleotide substitutions resulting in resistance-conferring amino acids in the encoded protein; and (3) losses of ancestral *Rdl* (Fig. 1a). Scale insects are primarily used by ladybirds as prey^39,40^. Our results suggest an evolutionary scenario in which the duplications and amino-acid-changing substitutions of *Rdl* in the ladybird ancestor conferred resistance to terpenoids used by scale insects as a chemical defence against natural enemies, which in turn contributed to the evolutionary radiation of ladybirds (Fig. 2a).

## Amino acid substitutions of *Rdl* contribute to terpenoid insensitivity

**Fig. 3:**
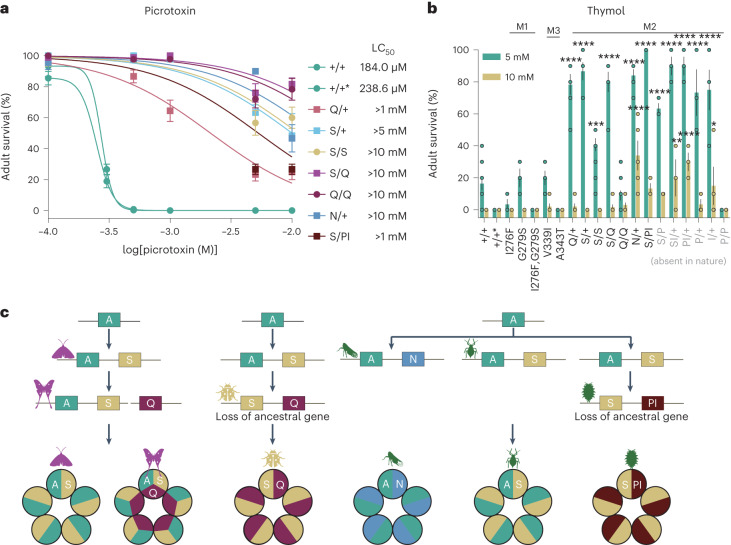
Amino acid replacements and duplications of *Rdl* contributing to picrotoxin and thymol insensitivity in gene-edited *D. melanogaster*. **a**,**b**, Adult survival of flies reared on diets containing picrotoxin (**a**) or thymol (**b**) among distinct lines. *Rdl* genotype: +/+, *w*^*1118*^ wild-type; +/+*, A2’A engineered control; S, A2ʹS; Q, A2ʹQ; N, A2ʹN; P, A2ʹP; and I, T6ʹI. log(inhibitor) versus response nonlinear fit was performed for **a**. One-way ANOVA with post hoc Bonferroni correction was used for **b** (Supplementary Table 16); mean ± s.e.m., *n* = 12, 3, 3, 3, 5, 3, 6, 6, 12, 6, 10, 5, 3, 3, 3, 3, 3, 4 and 6 biological replicates, and 10 females per replicate; *P < 0.05, **P < 0.01, ***P < 0.001, ****P < 0.0001. LC_50_, median lethal concentration. **c**, Model of stepwise duplications of *Rdl* in the insect lineages generates multiple subunits to form candidate homo- and hetero-pentameric ion channels conferring resistance to neurotoxic terpenoids. The line break between A, S and Q in Lepidoptera indicates that these are not on the same chromosome. Source data

**Extended Data Fig. 5:**
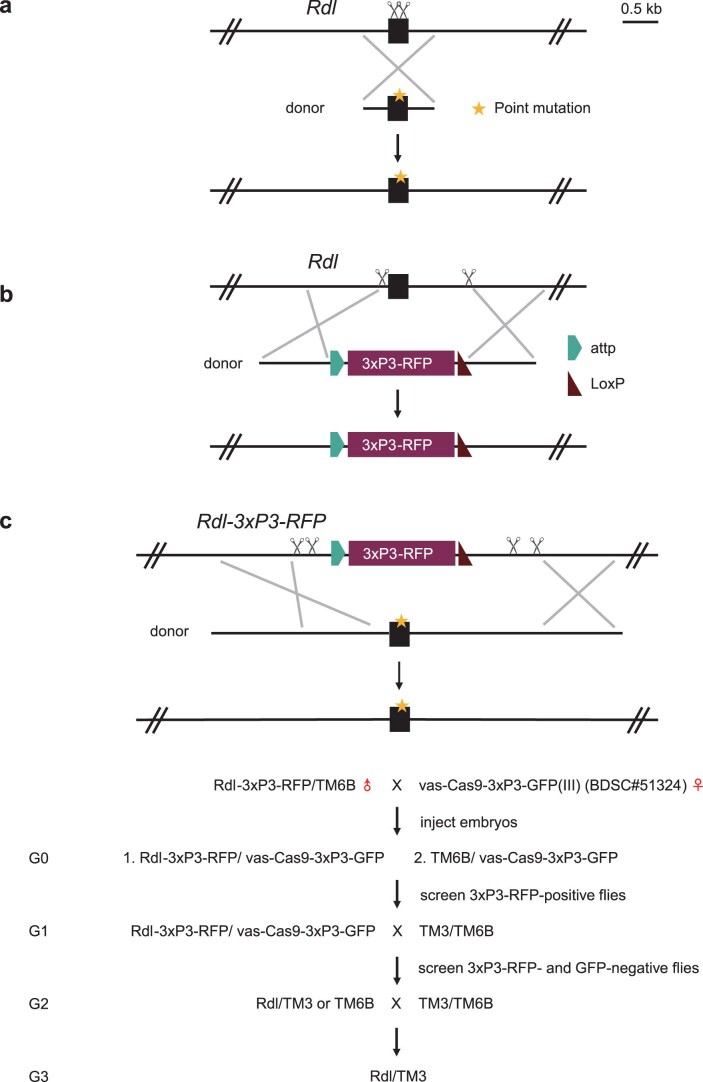
Schematic of the strategy for generating knock-in alleles of *Rdl* using a two-step CRISPR–Cas9 gene editing method. (**a**) The genomic region of *Rdl* was replaced with a 1000-bp homology arm carrying a point mutation through homologous recombination. (**b**) The genomic region of *Rdl* was first replaced with a 3xP3-RFP marker. (**c**) The 3xP3-RFP marker was then replaced with a homology arm carrying a point mutation. Yellow stars represent the point mutation. The crossing methods to generate knock-in lines are also shown. See also the generation of knock-in flies in Supplementary Tables 13–15 for further details. Methods generally follow from refs. ^41^ and ^48^.

To investigate whether the *Rdl* substitutions we identified provide resistance in vivo to both NCA and PAM terpenoids in whole organisms, and to assess their potential pleiotropic effects, we introduced them into the native *Rdl* gene of *D. melanogaster* mainly using a two-step CRISPR-Cas9 genome editing approach (Extended Data Fig. 5). To recapitulate the effects of gene duplication events and assess pleiotropic effects of different combinations of mutations, we also made a series of heterozygous lines to study the particular *Rdl* genotypes we found across a diversity of insect lineages (Fig. 3).

**Extended Data Fig. 6:**
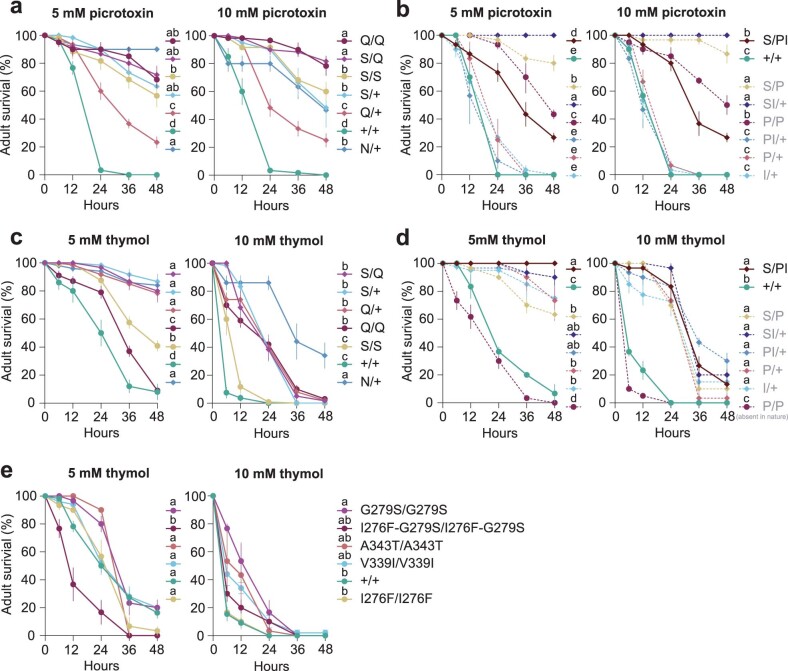
Amino acid replacements and duplications of *Rdl* show resistance to picrotoxin and thymol in gene-edited Drosophila melanogaster. (**a–e**) Adult survival of flies (see Methods for details) reared on diets containing picrotoxin (**a** and **b**) or thymol (**c–e**) among distinct lines. The mutations in M2 (**a–d**) and M1 and M3 (**e**) were tested. 5-7 old virgin female flies were gently introduced into vials containing different concentrations of picrotoxin or thymol. Genotype: +/+, wild-type; S, A2ʹS; Q, A2ʹQ; N, A2ʹN; P, A2ʹP; and I, T6ʹI. The log-rank (Mantel–Cox) test (Supplementary Table 16) was performed, mean ± SEM (a, n = 6, 6, 6, 6, 6, 6, and 3 biological replicates; b, n = 3, 3, 3, 3, 3, 3, 3, and 3 biological replicates; c, n = 6, 6, 6, 10, 12, 5, and 5 biological replicates; d, n = 3, 3, 3, 3, 3, 3, 4, and 6 biological replicates; e, n = 3, 3, 3, 5, 11, and 3 biological replicates and 10 females per replicate). Experimental groups denoted by ‘ab’ are not significantly different from either a or b groups. Source data

**Extended Data Fig. 7:**
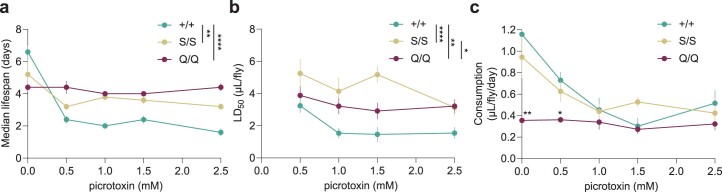
The mutational path of *Rdl* in the lepidopteran lineages associated with increased net diversification of species increases resistance to picrotoxin in gene-edited *Drosophila melanogaster*. (**a**) Estimation of mean lifespan in adult female flies in CAFE assays at a range of picrotoxin concentrations. Means were compared using two-way ANOVA and post hoc Tukey’s tests (Supplementary Table 16). (**b**) Estimation of LD50. Means were compared using two-way ANOVA and post hoc Tukey’s tests (Supplementary Table 16). (**c**) Estimation of feeding rates. Changes were compared to the wild-type flies. Means were compared using one-way ANOVA and post hoc Tukey’s tests (Supplementary Table 16). Genotype: +/+, wild-type; S, A2ʹS; and Q, A2ʹQ. Each data point represents the mean ± SEM of five biological replicates. *P < 0.05, **P < 0.01, ****P < 0.0001. Source data

We found that three lines (N, 6ʹI and P-6ʹI) were homozygous lethal, suggesting that these mutations exact high fitness costs. We then measured survival and performance using picrotoxin and thymol as representative NCA and PAM terpenoids, respectively, in different feeding assays. We found that S/+ (Ser/Ala) and S/S increased adult survival compared with the wild type when exposed to increasing concentrations of picrotoxin in the diet. Q/+ (Gln/Ala) also increased adult survival when fed on picrotoxin but were more sensitive to picrotoxin than S/+ and S/S. As expected, S/Q and Q/Q increased adult survival compared with the others at 10 mM picrotoxin (Fig. 3a and Extended Data Fig. 6a). Consistent with findings from a study that created ‘monarch flies’ that were fully resistant to dietary cardenolides^41^, the increased adult survival we observed was not due to a reduction in feeding rate or toxin ingestion (Extended Data Fig. 7). The N/+ (Asn/Ala) and S/PI (Ser/Pro-6ʹIle/Thr) genotypes also increased adult survival when fed picrotoxin (Fig. 3a and Extended Data Fig. 6b). In addition, we tested mutants in which *Rdl* genotypes (S/P, SI/+, PI/+, P/+, I/+ and P/P) are absent in the scale insect lineages. The homozygous P/P genotype increased adult survival upon picrotoxin exposure and the heterozygous S/P and SI/+ (S/+-6ʹI/+) both showed higher resistance than S/PI flies. However, three additional heterozygous lines, PI/+, P/+ and I/+, showed no significant resistance to picrotoxin (Extended Data Fig. 6b). In contrast, most of these lines were resistant to varying degrees upon exposure to thymol, except P/P, which was more sensitive than the wild type (Fig. 3b and Extended Data Fig. 6c,d). We also tested whether the amino acid substitutions in M1 and M3 regions provide resistance to thymol, but we found no evidence for an effect (Fig. 3b and Extended Data Fig. 6e). Our results indicate that amino acid replacements in the M2 of RDL encoded by the *Rdl* paralogues confer resistance to these exemplar NCA and PAM terpenoids in vivo, and the loss of the sensitive copy of *Rdl* (ancestral character state) may further enhance resistance.

Coexpression of *Rdl1* and *Rdl2* from moths can generate hetero-pentameric GABA receptors with intermediate sensitivity to dieldrin compared with their homo-oligomers^20^. More importantly, a duplication of the *Rdl* locus (2ʹA in one copy and 2ʹS in the other copy) resulted in functional and permanent heterozygosity with intermediate insecticide resistance in two natural *D. melanogaster* strains^42^. In agreement with this, we found that the diverse RDL subunits may form hetero-oligomers that result in variation in terpenoid resistance (Fig. 3a,b and Extended Data Fig. 6). Therefore, the duplications of *Rdl* in these insect lineages could generate a multiplicity of RDL subunits that form homo- or hetero-pentameric receptors. For example, two *Rdl* copies could result in 2^5^ (32) unique combinations and three copies could result in 3^5^ (243) unique combinations, resulting in potentially diverse functional properties and pharmacological characteristics (Fig. 3c). Our findings suggest that subtle genotypic differences in *Rdl* belie a far more complex range of phenotypic possibilities at the level of the pentameric receptor in this system. However, it should be noted that the subunit stoichiometry of any insect GABA receptor is unknown and the actual number of receptor subtypes could be fewer than the above calculations. Further physiological and structure–function studies of the GABA receptor are needed to evaluate the biological significance of these findings.

## Amino acid replacements of *Rdl* are associated with fitness costs

**Fig. 4:**
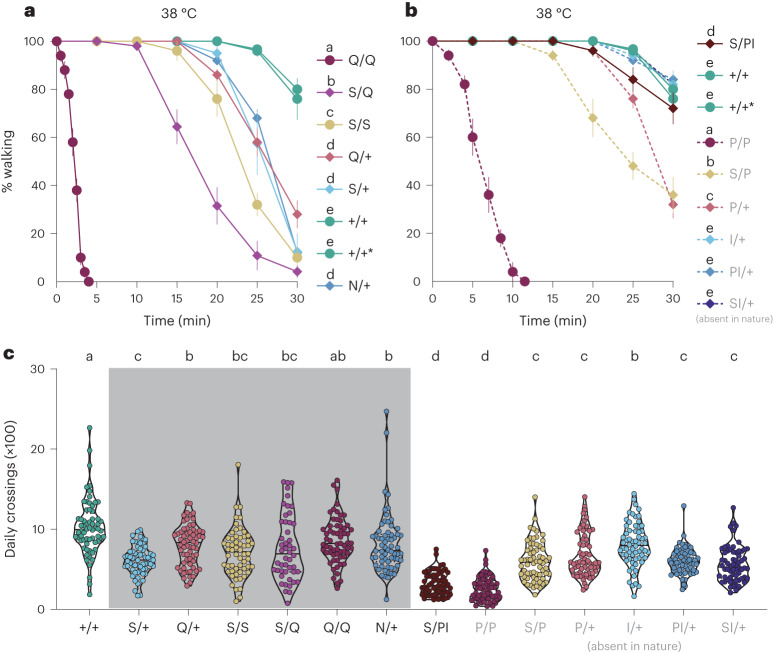
Amino acid replacements and duplications of *Rdl* are associated with fitness costs in *D. melanogaster*. **a**,**b**, The percentage of flies observed to be walking when exposed to high temperature for 30 min. Repeated-measures ANOVA (general linear model) and post hoc Bonferroni correction (Supplementary Table 16) for **a**, *n* = 5, 5, 5, 5, 4, 5, 3 and 5 trials; for **b**, *n* = 5, 5, 3, 5, 5, 5, 5, 5 and 5 trials, and 10 females per trial, mean ± s.e.m. **c**, Daily crossing activity. One-way ANOVA and post hoc Bonferroni correction (Supplementary Table 16) were used for statistical analysis; mean ± s.e.m., *n* = 64, 58, 63, 62, 61, 64, 54, 64, 62, 64, 64, 64, 64 and 59. Genotypes as in Fig. 3. Letters indicate significant differences among genotypes and experimental groups denoted by ‘ab’ are not significantly different from either a or b groups. Source data

**Extended Data Fig. 8:**
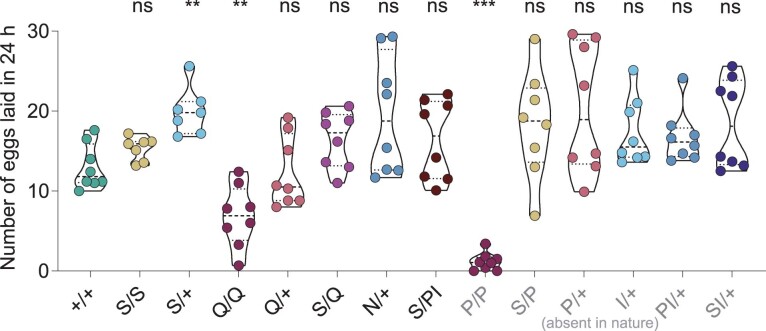
Effects of *Rdl* mutations on eggs laid in gene-edited *Drosophila melanogaster*. S/S flies showed normal egg-laying behavior, while Q/Q and P/P had serious deleterious effects. However, deleterious effects of these amino acid replacements were ameliorated through the formation hetero-pentameric GABA receptors. Comparisons to wild-type flies are shown. n = 8, 7, 7, 8, 8, 8, 8, 8, 8, 8, 8, 8, 8, and 8 trials, 10 females/trial, p values, ns, not significant (P > 0.05); **P < 0.01; ***P < 0.001. Means were compared using Kruskal-Wallis and post hoc Mann–Whitney U tests, mean ± SEM. Genotype: +/+, wild-type; S, A2ʹS; Q, A2ʹQ; N, A2ʹN; P, A2ʹP; I, T6ʹI. Source data

**Extended Data Fig. 9:**
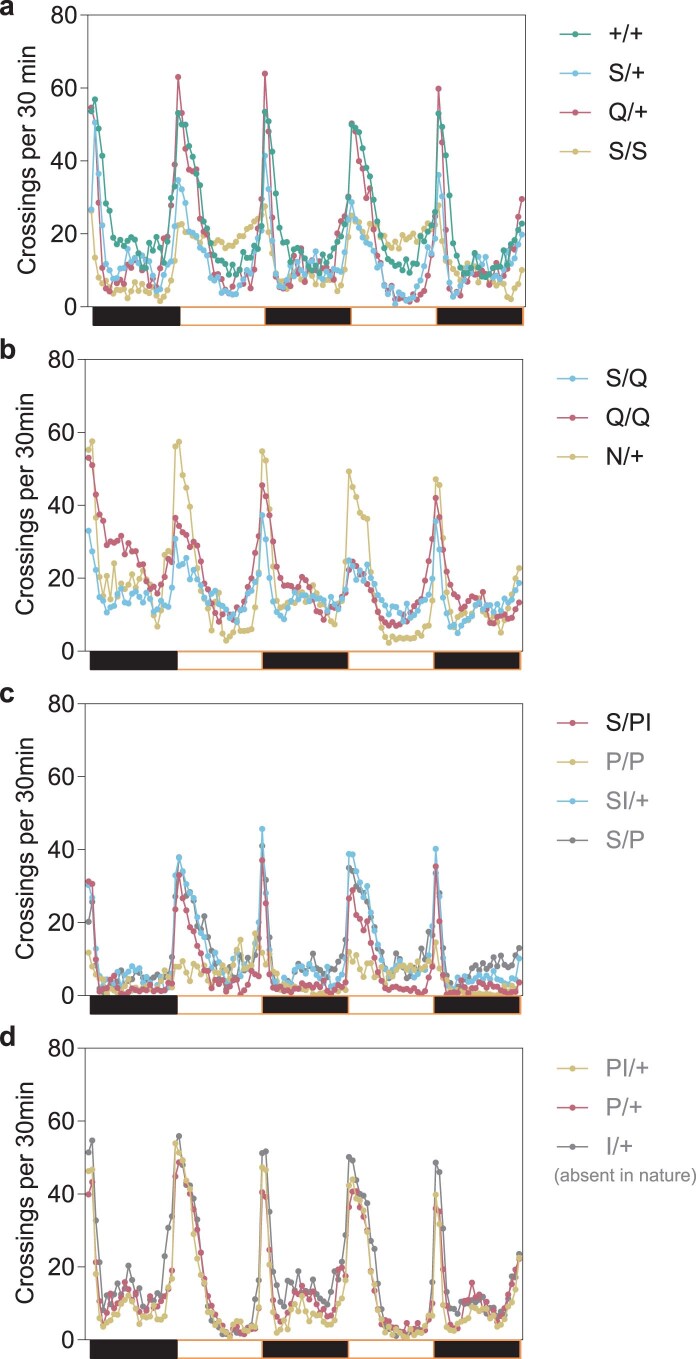
Daily activity across adult *Drosophila melanogaster* of different engineered genotypes. **a**-**d**, 5-7 old female flies of different genotypes were gently introduced into each tube containing 2% agarose and 5% sucrose diets, the other end was sealed with a cotton plug. Black and white bars represent a 12-h light: dark cycle. Daily cross activity test in a 2-day window in the yellow box. mean ± SEM, n = 24-32. Genotype: +/+, wild-type; S, A2ʹS; Q, A2ʹQ; N, A2ʹN; P, A2ʹP; I, T6ʹI. Source data

Given the essential role of GABA receptors in the nervous system and the high degree of RDL sequence conservation across animals, mutations that confer resistance may impair the ancestral function. We addressed this by measuring potential fitness costs in the *Drosophila* mutants we created. In comparison with control flies, Q/Q and P/P genotypes decreased egg laying of females, while other lines showed no measured defects (Extended Data Fig. 8). Previous studies found that the A2ʹS mutation causes a temperature-sensitive phenotype in *Drosophila*^42,43^, so we also performed a heat-shock test across our lines. We found that S/S genotype flies began to exhibit paralysis behaviour after exposure to 38°C for 15 min. Notably, Q/Q homozygote flies became paralysed more rapidly and were completely unable to move after 5 min (Fig. 4a and Supplementary Video 1). By contrast, S/+ and Q/+ heterozygous flies showed higher thermal resistance compared with S/S and Q/Q genotype flies, respectively. The S/Q heterozygote flies displayed an intermediate heat-shock sensitivity that was in-between the levels found in the S/S and Q/Q homozygous flies (Fig. 4a). The N/+ heterozygous flies also exhibited defects compared to wild-type flies (Fig. 4a). Importantly, S/PI heterozygous flies displayed higher temperature tolerance than S/P, P/P or P/+ flies, suggesting that the pleiotropic costs of 2ʹP are rescued to some degree by the 6ʹI mutation (Fig. 4b). Finally, we measured locomotory behaviour using an automated monitoring system and found that all lines carrying engineered mutations exhibited a decrease in locomotion, particularly the S/PI and P/P lines (Fig. 4c and Extended Data Fig. 9). The severe deleterious effects of S/PI on movement may be tolerable in the scale insect radiation since the immature life stages most susceptible to predation and parasitism are slow moving or immobile. Overall, these results show that the coexistence of multiple *Rdl* copies ameliorates antagonistic pleiotropy while maintaining resistance to diverse terpenoids.

## Discussion

Identifying the molecular mechanisms arising from coevolution between plants and herbivorous insects is difficult because it involves determining how sets of species have coadapted through vast expanses of time^1,3,4,44,45^. The repeated evolution of resistance to synthetic NCA insecticides provides a useful window into how insects responded to repeated bursts of novel terpenoid insecticide production across deep time as ancient plants diversified and their terpene synthase repertoire expanded via genome duplication events^46^.

Amino acid substitutions and gene duplication events repeatedly evolved in the insect GABA receptor subunit gene *Rdl* through selection for cyclodiene and phenylpyrazole resistance over the past 70 yr^14,16,47^. The A2ʹS and A2’N substitutions, which confer resistance to these insecticides, are now globally widespread in diverse arthropod lineages^11,25^. Remarkably, the same mutations and parallel gene duplication events became fixed in four different herbivorous lineages and one predator lineage over the past 300 Myr, indicating that the adaptive path is repeatable and therefore predictable under similar selective pressures^41,48^.

These molecular evolutionary changes in *Rdl* were associated with host plant switching events across herbivorous insects and an increased net diversification rate in the Lepidoptera, one of the most diverse insect orders (Fig. 2). However, it remains unclear why Coleoptera and Hemiptera lack a signal of increased diversification rate associated with the evolution of resistant GABA receptors. The Lepidoptera experienced the most recent *Rdl* duplications, which occurred ~94 Ma, while *Rdl* duplications in the Coleoptera and Hemiptera occurred over 180 Ma. This, together with the long internal branches and likely high extinction rates in the Coleoptera and Hemiptera over deep time, may have obscured signals of a diversification rate increase associated with *Rdl* duplication. Alternatively, adaptive evolution of *Rdl* did not spur diversification in these two orders.

Fossil evidence suggests that the stem lineage of aphids, which originated in the Triassic, fed on gymnosperm hosts^49–51^. Our results provide a conceptual model of how this may have proceeded at the genetic and protein receptor levels in several major herbivorous insect lineages as well as the ladybirds, a major predator of sternorrhynchan insects (aphids, scale insects, psyllids and whiteflies). Amino acid replacements at position 2’ occurred in all duplicated copies and these RDL subunits may form heteromeric ion channels that confer resistance to terpenoids while minimizing fitness costs through the amelioration of antagonistic pleiotropy. We hypothesize that such properties would provide persistent fitness advantages in ancient herbivore lineages that encountered an increasing diversity of terpenoid toxins targeting the GABA receptor through both NCA and PAM mechanisms.

Unlike other insect lineages with two *Rdl* copies, the macroevolutionary patterns underlying terpenoid resistance in moths carrying multiple copies may reflect two peaks on an adaptive landscape. Given that the *Rdl3* copies carrying 2’Q confer higher resistance to picrotoxin than the 2’S-harbouring *Rdl2* (Fig. 3a and Extended Data Fig. 6a), a stepwise evolutionary path was taken by the moth radiation. The initial duplication that generated *Rdl2* may have provided an advantage that spurred early host shifts to gymnosperms. The origin of the angiosperms and the rapid diversification of core eudicots provided more diverse host plants, terpenoid synthases and terpenoids by the early Cretaceous. However, many lepidopteran clades are restricted to plants from particular angiosperm lineages^52^. The second duplication event that generated *Rdl3* may have facilitated an escape from ancestral host plant lineage of moths and exploitation of other core eudicots, which then led to a second major adaptive radiation. This hypothesis is congruent with patterns of host use and diversity of the superfamily Noctuoidea, the most species rich of any superfamily, containing nearly one-third of all lepidopteran species^53^.

Core eudicots produce a greater diversity of PAMs compared with gymnosperms. Structures of GABA_A_ receptors in complex with PAMs, such as benzodiazepines, suggest that the binding pocket includes the M3–M1 interfaces along with the 15’ residue in M2 (ref. ^27^). However, we found that the substitutions in M1 and M3 have no effect on PAM resistance (Fig. 3b and Extended Data Fig. 6e), but the substitutions at the 2’ position in M2 confer resistance to the PAM thymol (Fig. 3b). These M2 substitutions both directly affect the binding at the NCA drug binding site and also allosterically destabilize the PAM drug preferred desensitized state as previously described^15^.

Gene duplications and amino acid substitutions in *Rdl*, together with the pre-mRNA A-to-I editing and alternative splicing^15^, may also confer structural diversity to the pentameric GABA receptor complex, yet the mechanistic details of how homo- or hetero-pentameric receptors assemble are unclear. Such cryptic GABA receptor diversity would facilitate the maintenance of terpenoid resistance but also relax constraints imposed by resistance substitutions over deep time.

In summary, we found evidence that the convergent evolution of *Rdl* conferred resistance to neurotoxic plant terpenoids associated with herbivore host shifts, higher net diversification in moths and butterflies, and an adaptive radiation of a predatory beetle family. Our results support a cascading model of coevolution in which the increasing complexity of terpenoid defensive cocktails in plants was followed by the evolution of resistant GABA receptors in the herbivores and their predators. Thus, terpenoids and resistant GABA receptors could be one example of coupled key innovations that have contributed to the adaptive radiation of insects and plants at macroevolutionary timescales. A scenario similar to the one that unfolded over 300 Myr of coevolution has also played out across a single human generation, as insects adapted to synthetic insecticides that functionally mimic natural neurotoxic terpenoids.

## Methods

### Identification of *Rdl* genes in insects and phylogenetic analyses of RDL

**Extended Data Fig. 10:**
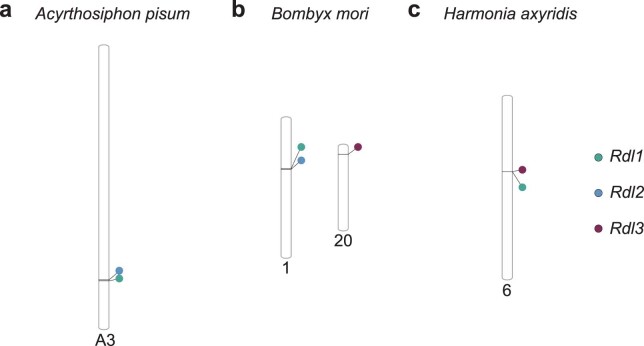
The duplicated copies of *Rdl* are mapped on chromosomes of *Acyrthosiphon pisum* (Hemiptera) (a), *Bombyx mori* (Lepidoptera) (b), and *Harmonia axyridis* (Coleoptera) (c), respectively. Blue, green, and red circles represent *Rdl1*, *Rdl2*, and *Rdl3*, respectively.

To identify *Rdl* genes in insects, we performed a two-step analysis: (1) we used *D. melanogaster*, *Bombyx mori*, *Chilo suppressalis* and *Acyrthosiphon pisum Rdl* genes as queries to perform BLASTp and TBLASTn searches against genomes and transcriptomes from GenBank, AphidBase, InsectBase 2.0, Fireflybase, DRYAD, Lepbase and GigaDB, because the *Rdl* genes from these species have already been confirmed^18,20,22^; (2) we verified the candidate genes by BLASTp again without a species limit as we have shown before^54,55^. We took all the candidate *Rdl* genes that were reciprocal best hits with the *D. melanogaster* and *B. mori Rdl* genes and then renamed them (Supplementary Table 1). The *Rdl* copies were mapped on chromosomes in three representative species (Extended Data Fig. 10).

To estimate the evolutionary relationships of *Rdl* paralogues among different orders, we used IQ-TREE (v.1.6.12) to build a maximum likelihood (ML) phylogenetic tree using an amino acid alignment of RDL^56^. To create the alignment, nucleotide sequences were predicted and translated to proteins by NCBI ORFfinder (https://www.ncbi.nlm.nih.gov/orffinder/). The four *Varroa destructor* (Arachnida) RDL amino acid sequences were used as the outgroup. We then selected the RDL sequences of more than 360 amino acids in Hemiptera, Coleoptera, Hymenoptera and Lepidoptera and estimated the phylogeny. We report the *Rdl* sequences used in our analysis in Supplementary Table 1. Protein sequence alignment was then performed using MUSCLE in MEGA X^57,58^. The best-fit model was VT+I+G4 estimated using ModelFinder^59^, and an ML tree inference was subsequently conducted in IQ-TREE with default parameters. The tree with the best log-likelihood was selected as the tree for further analysis.

We found that duplications of *Rdl* are always associated with amino acid substitutions at site 2′. NCA insecticides, such as dieldrin and fipronil, also act on this site and many agricultural pests have already evolved segregating mutations at position 2′, which confer resistance to insecticides^11^. In light of this initial finding, we decided not to analyse the 2′ substitutions in the absence of duplication events. In particular, we did not find parallel amino-acid-changing mutations in sister groups without *Rdl* duplications, implying that these may have arisen recently and are associated with insecticide resistance. For example, in Thripidae, we observed that *Rdl* sequences from *Frankliniella occidentalis*, *Thrips tabaci,* and *T. palmi* have the A2′S mutations, whereas those from *F. cephalica* and *Aptinothrips rufus* do not. Notably, *F. occidentalis* from Utah (USA, accession number: GCYR01013327.1) does not have A2’S *Rdl* mutations, whereas *F. occidentalis* from Yangzhou has an A2′S mutation associated with insecticide resistance (China, accession number: MH249048.1), suggesting that the *F. occidentalis* from Yangzhou recently evolved resistance to insecticides via target site insensitivity.

### Species tree

To further study the duplication histories of *Rdl* across insects and conduct phylogenetically informed diversification studies, we estimated a species tree of all lineages screened for *Rdl* copy number variation and resistance substitutions using 1,123 single-copy orthologues extracted using the BUSCO pipeline and the insecta_odb10.2019-11-20 marker set^60^. We used genome and transcriptome assemblies downloaded from GenBank, AphidBase, InsectBase 2.0, Fireflybase, DRYAD, Lepbase and GigaDB. Amino acid sequences of each orthologue were aligned across all genome and transcriptome assemblies in which the orthologue was complete. We aligned amino acid sequences in MAFFT^61^ and then inferred ML trees for each orthologue in IQ-TREE after automatic best-fit model selection^56^. These gene trees were then used to infer a species tree in ASTRAL^62^.

A family-level phylogeny was then estimated on the basis of previously published sources (see Supplementary Table 2 for references), in which we pruned the taxa to a single species representing each family. Three orders, Hemiptera, Coleoptera, and Lepidoptera, were used for this analysis and the tree was rooted with sequences from Dermaptera (*Diplatys* sp.), Plecoptera (*Moselia infuscata*), and Orthoptera (*Myrmecophilus* sp.) (Supplementary Table 1).

### Diversification

We applied sister-clade analysis using the ‘richness.yule.test’ function in the R package ape to test whether ancestral shifts in *Rdl* copy number are associated with shifts in diversification rate given extant species richness. We used stem ages taken from primary phylogenetic studies (see Supplementary Table 2 for references) and species richness estimates from the Global Biodiversity Information Facility^63^. *Rdl* copy number is a good proxy for insecticide resistance because duplications of *Rdl* are tightly associated with resistance substitutions. Sister-clade analysis is a standard approach to understanding asymmetry on a phylogenetic tree without requiring fully sampled phylogenies^64^, which is useful in this case because our sampling of the insect phylogeny is incomplete. Although sister-clade analysis has a long history, biases exist in comparisons between sister clades with different crown ages^65^. The reason for this bias is that younger clades are more likely to be species poor and contain the derived trait because the waiting time given by the stem branch is long. In our analysis, we used stem ages and model species richness probabilities as a Yule process^66^. We conducted the sister-clade analysis using six sister-clade pairs and tested for the contribution of any one pair by conducting additional sister-clade analyses with one pair removed.

Next, we estimated net diversification rates using the ‘bd.ms’ function in the R package geiger^67^ under different extinction rate scenarios (0% extinction, 50% extinction relative to speciation rate, 90% extinction relative to speciation rate). Stem ages and species richness estimates were taken as above.

### Ancestral-state reconstruction of feeding states

Terpenoids targeting GABA receptors were collated from previous studies on the basis of pharmacological assays (Supplementary Table 4). The distribution of these terpenoids was primarily taken from ref. ^68^ (Supplementary Table 5). For the family-level feeding preference of Hemiptera, we calculated the proportion and the number of species that feed on gymnosperms (Supplementary Table 6). Data were taken from ref. ^69^. For the family-level feeding preference of Lepidoptera, we calculated the proportion and the number of species that feed on terpenoid-containing plants (Supplementary Table 7). Data were taken from the ref. ^70^. Any family in which <3 species were excluded for family-level ancestral-state reconstructions and in which there is a >1% result indicates that there is strong evidence of feeding states. The dietary preferences of the ladybird species were based on previous studies (Supplementary Table 12).

We conducted ancestral-state reconstructions on the family-level phylogeny (the species tree) using PastML with default parameters^71^. In addition, ML and marginal posterior probabilities approximation methods were used to assess the state posteriors on a node. We visualized the ancestral states and phylogenetic trees using the Interactive Tree Of Life^72^.

### Associations of RDL M2 sequences and feeding states

We used TraitRateProp to detect the associations between genotypes and phenotypes across the phylogeny^73^. We focused on the association between the M2 sequences and feeding states. A rooted ultrametric tree, RDL amino acid alignments and trait states were used for the analysis. The rooted ultrametric tree was constructed on the basis of previously published sources (see Supplementary Table 2 for references).

### Fly strains

*Drosophila melanogaster* flies were reared on conventional cornmeal-agar-molasses medium at 60% ± 10% humidity and 25 ± 1 °C with the day and night periods of the 12 h:12 h light:dark cycle. The light was set at 07:00. The *w*^*1118*^ (#5905) strain was used as the wild-type strain in this study. The following strains were obtained from the Bloomington Stock Center (Indiana University): *Rdl*^*MDRR*^ (*Rdl*^*A2*′*S*^, #1675), *vas-Cas9* (#51323, #51324), and *nanos-Cas9* (ref. ^74^).

### Generation of knock-in flies

To generate the *Rdl* knock-in lines, we used a two-step CRISPR (clustered regularly interspaced short palindromic repeats)-Cas9 method relying on homology-directed repair (HDR). We designed single guide (sg)RNAs using E-CRISP (http://www.e-crisp.org/E-CRISP/) and CHOPCHOP (https://chopchop.cbu.uib.no/)^75,76^. The sgRNAs were synthesized by GenScript and then subcloned into the PCFD5 vector for expression.

We used two specific approaches to generate *Rdl* knock-in lines (Extended Data Fig. 5 and Supplementary Tables 13–15). First, since many terpenoids, including picrotoxin and thujone, and synthetic insecticides, act on the 2′, 6′ and 9′ residues of M2, we reasoned that 2′ mutations could confer resistance to these chemicals. Therefore, we synthesized a single 1,000 bp homology template from *D. melanogaster* genomic DNA containing A2′Q or A2′P mutation and subcloned it with EcoRI and HindIII sites into the pUC57 vector. Following embryo injection procedures described below, the knock-in flies were identified through a screen in which we reared adult flies on a picrotoxin diet and genotyped *Rdl* using Sanger sequencing of PCR amplicons from the surviving flies. We used this method to generate the *Rdl*^*A2*′*Q*^ strain.

Second, we synthesized two 1 kb genomic DNA fragments obtained from *D. melanogaster* genomic DNA corresponding to the M2 sequences of the *Rdl* gene as homology arms and subcloned them into the pBSK-attP-3xP3-RFP-loxP vector^77^ to generate the *Rdl*^3*x*P3RFP^ strain. Next, the attP-3xP3-RFP-loxP sequence was replaced with each of the point mutation alleles in *Rdl* through homologous recombination, generally following ref. ^41^. Candidate 3xP3-RFP-positive or -negative flies were verified by PCR of the targeted region followed by Sanger sequencing. We used this method to generate the *Rdl*^3*x*P3RFP^, *Rdl*^*A2*′*A*^, *Rdl*^*A2*′*N*^, *Rdl*^*A2*′*P*^, *Rdl*^*T6*′*I*^, *Rdl*^*A2*′*P-T6*′*I*^, *Rdl*^*I276F*^, *Rdl*^*G279S*^, *Rdl*^*I276F-G279S*^, *Rdl*^*V339I*^, and *Rdl*^*A343T*^ strains. Note that *Rdl*^*A2*′*A*^ is an engineered control strain containing synonymous codon mutations at PAM sites, which was created to account for potential pleiotropic or off-target effects of the two rounds of CRISPR-Cas9 mediated HDR.

To generate these knock-in lines, a plasmid mixture with the donor vector and sgRNAs was injected into *vas-Cas9* (51323), *nos-cas9* (78781) or *Rdl*^3P3RFP^/*vas-Cas9* (51324) embryos, as shown in Extended Data Fig. 5 and Supplementary Table 13. The *vas-Cas9* (51324) strain carrying the 3xP3-GFP marker on chromosome 3 was crossed with *Rdl*^3*x*P3RFP^ to produce heterozygous flies as G_0s_ for embryo injections. Typically, 250–300 embryos were injected (UniHuaii) and RFP-positive flies were screened under a fluorescence microscope and crossed with double-balanced flies (*TM3/TM6B*). The G_1s_ were then screened for RFP- and GFP-negative flies, which were crossed with double-balanced flies. The G_2s_ were verified by PCR and Sanger sequencing as above.

### Bioassays

Picrotoxin (Tokyo Chemical Industry, C0375) was resuspended and serially diluted in 100% acetone (Sinopharm, 10000418) solution. Thymol (Aladdin, T104426) was resuspended in 100% acetone and serially diluted in 50% v/v acetone/50% deionized water solution. All compounds were freshly prepared before each assay. Then, 100 µl of picrotoxin or thymol solution was added to the surface of 12 × 78 mm *Drosophila* vials containing 900 µl 2% (w/v) agarose and 5% (w/v) sucrose. We prepared the vials 20–24 h before the assays to allow for the complete distribution of the chemicals and the evaporation of the solution as previously described^78^. Ten mated female flies aged 5–7 days old were gently introduced into the vials and reared at 25 °C, 30% humidity and a 24 h (12 h:12 h dark:light) period. Adult survival was monitored for 48 h.

### Behavioural assays

For egg-laying assays, 10 females and 5 males were grouped and reared on conventional cornmeal-agar-molasses medium in single vials. After 5–7 days, the female flies were transferred to 35 mm tissue culture dishes containing fresh food for 24 h, and the number of eggs was manually counted under a stereo microscope.

For temperature sensitivity assays, 10 female flies aged 5–7 days old were gently introduced into new empty glass fly vials by aspiration. The flies were exposed to a 38 °C temperature and recorded with a video camera for 30 min. The percentage of flies awake in each tube was measured every 30 s. The knock-in lines exhibited paralysis behaviour when exposed to the high temperature, in which the animal lies on its dorsum and is unable to locomote.

For locomotion assays, locomotor behaviour was performed as previously described^54^. Individual 5- to 7-day-old virgin female flies loaded into tubes with 2% (w/v) agarose and 5% (w/v) sucrose were monitored using the *Drosophila* Activity Monitoring System (DAMS, Trikinetics) in 24 h light/dark cycles. The flies were allowed to adapt to the new environment for 1 day and data were collected in 30 min bins. Average daily activities were measured on the basis of a 2 day window.

### Molecular docking

The human α1β3γ2 and α1β2γ2 GABA_A_ receptors and picrotoxin and propofol-bound crystal structures (PDB: 6HUG and 6X3T) provided homology templates for building a homo-pentameric model of the *Drosophila* RDL receptor^27,31^. Molecular Operating Environments (MOE, 2015.10) was used as previously described^54^. The best model was chosen on the basis of the scoring values and evaluated using the UCLA-DOE server and Ramachandran plots. The models of RDL mutations were generated using Swiss-PdbViewer to introduce the amino acid substitutions and minimize the energy of the resulting structures^79^. The pore diameters of the models were calculated using the HOLE in WinCoot^80^. The MOE-Dock programme was used for docking with default parameters. 3D protonation was added, water molecules were deleted, and the energy of the models and ligands was minimized. The ligand was kept flexible during molecular docking calculations. A lower binding-free energy structure, which indicates a better interaction between the model and ligand, was chosen for the analyses.

### Statistical analysis

All statistical analyses were performed using GraphPad Prism 7 (GraphPad software). The adult survival curves were analysed using log(inhibitor) versus response nonlinear fit and the log-rank (Mantel–Cox) test, and *P* values were compared to the Bonferroni correction values: *α*_adjusted_ = 0.05/(*n*(*n*−1)/2), where *n* is the number of groups. Repeated-measures analysis of variance (ANOVA) and post hoc Bonferroni correction were performed for temperature sensitivity assays. One-way ANOVA and post hoc Bonferroni correction were used for locomotion assays. Finally, Kruskal–Wallis and post hoc Mann–Whitney *U* tests were performed for egg-laying assays. Details on the other statistical methods are reported in the figure legends.

### Reporting summary

Further information on research design is available in the Nature Portfolio Reporting Summary linked to this article.

## Supplementary information

Supplementary Information Legends for Supplementary Tables 1–16 and Video 1, and References.

Reporting Summary

Supplementary Tables Supplementary Tables 1–16.

Supplementary Data 1 Net diversification rates spanning all lineages in this study.

Supplementary Data 2 RDL alignment for Extended Data Fig. 1.

Supplementary Video 1 Adult *Drosophila melanogaster* flies exposed to 38 °C temperature for 30 min.

## Data Availability

Net diversification rates and sequence alignments are available as Supplementary Data. Source data are provided with this paper.

## Source data

Source Data Fig. 3 Statistical source data.
Source Data Fig. 4 Statistical source data.
Source Data Extended Data Fig. 6 Statistical source data.
Source Data Extended Data Fig. 7 Statistical source data.
Source Data Extended Data Fig. 8 Statistical source data.
Source Data Extended Data Fig. 9 Statistical source data.
