# Supplementary material for: Convergent resistance to GABA receptor neurotoxins through plant–insect coevolution
Source: Nat Ecol Evol. 2023 Jul 17;7(9):1444–56. doi: 10.1038/s41559-023-02127-4 (PMC10482695; doi:10.1038/s41559-023-02127-4)
Supplement: Supplementary file 1 — Legends for Supplementary Tables 1–16 and Video 1, and References. [file 41559_2023_2127_MOESM1_ESM.pdf]

nature ecology & evolution

Article https://doi.org/10.1038/s41559-023-02127-4

Convergent resistance to GABA receptor neurotoxins through plant–insect coevolution

In the format provided by the authors and unedited

SUPPLEMENTAL INFORMATION

Legends for Supplementary Tables 1-16

Supplementary Table 1.
List of *Rdl* sequences and accession numbers used in this study.

Supplementary Table 2.
References for the phylogenetic relationships of taxa used in this study.

Supplementary Table 3.
Mutations in *Rdl* associated with cyclodiene or phenylpyrazole insecticide resistance.

Supplementary Table 4.
Known natural terpenoids that target insect GABA and mammalian $GABA_A$ receptors.

Supplementary Table 5.
Distribution of terpenoids targeting GABA receptor at the plant family level (mainly from Dr. Duke's Phytochemical and Ethnobotanical Databases).

Supplementary Table 6.
Number Hemiptera species that feed on gymnosperms that produce terpenoids targeting GABA receptors (data from The Database of British Insects and their Foodplants[69]).

Supplementary Table 7.
Numbers of Lepidoptera species that feed on plants that produce terpenoids targeting GABA receptors (data from the HOSTS-A Database of the World's Lepidopteran Hostplants).

Supplementary Table 8.
Sister lineages used for sister-clade analysis.

Supplementary Table 9.
Results of generalized linear models of the effect of *Rdl* copy number on net diversification rate under different extinction scenarios. Bold values with a yellow background indicate statistical significance based on p-values calculated using the glm function (two-tailed test) in base R.

Supplementary Table 10.

PGLS results for lambda = 0.1. Bold values with a yellow background indicate statistical significance based on p-values calculated using the pgls function (two-tailed test) in the R package caper.

Supplementary Table 11.

PGLS results for lambda = 0.5. Bold values with a yellow background indicate statistical significance based on p-values calculated using the pgls function (two-tailed test) in the R package caper.

Supplementary Table 12.

Diet preferences of the ladybird species used in this study.

Supplementary Table 13.

Wild-type and Crispr-Cas9 gene-edited *Drosophila melanogaster* knock-in lines generated and used in this study.

Supplementary Table 14.

Sequences of sgRNAs for generation of *Drosophila melanogaster* knock-in lines.

Supplementary Table 15.

RDL M2 region (exon 7) sequences of wild-type and knock-in *Drosophila melanogaster* lines (engineered point mutations are highlighted in yellow background)

Supplementary Table 16.

Statistical analyses

Supplementary Video

Supplementary Video 1.

Adult *Drosophila melanogaster* flies exposed to 38°C temperature for 30 min.

Genotype: A, wild-type; S, A2'S; Q, A2'Q. The movie was speeded up 5x.

Supplemental References

81. Misof, B. *et al.* Phylogenomics resolves the timing and pattern of insect evolution. *Science* **346**, 763–767 (2014).

82. Feindt, W., Oppenheim, S. J., DeSalle, R., Goldstein, P. Z. & Hadrys, H. Transcriptome profiling with focus on potential key genes for wing development and evolution in *Megaloprepus caerulatus*, the damselfly species with the world's largest wings. *PLoS One* **13**, 1–17 (2018).

83. Simon, S. *et al.* Comparative transcriptomics reveal developmental turning points during embryogenesis of a hemimetabolous insect, the damselfly *Ischnura elegans*. *Sci. Rep.* **7**, 13547 (2017).

84. Simon, S., Blanke, A. & Meusemann, K. Arthropod structure & development reanalyzing the Palaeoptera problem-the origin of insect flight remains obscure. *Arthropod Struct. Dev.* **47**, 328–338 (2018).

85. Wipfler, B. *et al.* Evolutionary history of Polyneoptera and its implications for our understanding of early winged insects. *Proc. Natl. Acad. Sci. U. S. A.* **116**, 3024–3029 (2019).

86. South, E. J. *et al.* Phylogenomics of the north American Plecoptera. *Syst. Entomol.* **46**, 287–305 (2021).

87. Prasad, M. P. *et al.* Transcriptional expression changes during compensatory plasticity in the terminal ganglion of the adult cricket *Gryllus bimaculatus*. *BMC Genomics* **22**, 742 (2021).

88. Oppert, B., Perkin, L. C., Lorenzen, M. & Dossey, A. T. Transcriptome analysis of life stages of the house cricket, *Acheta domesticus*, to improve insect crop production. *Sci. Rep.* **10**, 3471 (2020).

89. Kasumovic, M. M., Chen, Z. & Wilkins, M. R. Australian black field crickets show changes in neural gene expression associated with socially-induced morphological, life-history, and behavioral plasticity. *BMC Genomics* **17**, 827 (2016).

90. Foquet, B., Castellanos, A. A. & Song, H. Comparative analysis of phenotypic plasticity sheds light on the evolution and molecular underpinnings of locust phase polyphenism. *Sci. Rep.* **11**, 11925 (2021).

91. Konopová, B., Buchberger, E. & Crisp, A. Transcriptome of pleuropodia from locust embryos supports that these organs produce enzymes enabling the larva to hatch. *Front. Zool.* **17**, 4 (2020).

92. Yuan, H., Chang, H., Zhao, L., Yang, C. & Huang, Y. Sex- and tissue-specific transcriptome analyses and expression profiling of olfactory-related genes in *Ceracris nigricornis* Walker (Orthoptera: Acrididae). *BMC Genomics* **20**, 808 (2019).

93. Zhang, W. *et al.* Central nervous system responses of the oriental migratory, *Locusta migratoria manilensis*, to fungal infection. *Sci. Rep.* **7**, 10340 (2017).

94. Liessem, S., Ragionieri, L., Neupert, S., Büschges, A. & Predel, R. Transcriptomic and neuropeptidomic analysis of the stick insect,

Carausius morosus. *J. Proteome Res.* **17**, 2192–2204 (2018).

95. Kaku, K. & Matsumura, F. Identification of the site of mutation within the M2 region of the GABA receptor of the cyclodiene-resistant German cockroach. *Comp. Biochem. Physiol. Part C Comp.* **108**, 367–376 (1994).

96. Bucek, A. *et al.* Evolution of termite symbiosis informed by transcriptome-based phylogenies. *Curr. Biol.* **29**, 3728-3734.e4 (2019).

97. Johnson, K. P. *et al.* Phylogenomics and the evolution of hemipteroid insects. *Proc. Natl. Acad. Sci. U. S. A.* **115**, 12775–12780 (2018).

98. Rotenberg, D. *et al.* Genome-enabled insights into the biology of thrips as crop pests. *BMC Biol.* **18**, 142 (2020).

99. Jouraku, A. *et al.* T929I and K1774N mutation pair and M918L single mutation identified in the voltage-gated sodium channel gene of pyrethroid-resistant *Thrips tabaci* (Thysanoptera: Thripidae) in Japan. *Pestic. Biochem. Physiol.* **158**, 77–87 (2019).

100. Chen, W. *et al.* The draft genome of whitefly Bemisia tabaci MEAM1, a global crop pest, provides novel insights into virus transmission, host adaptation, and insecticide resistance. *BMC Biol.* **14**, 110 (2016).

101. Saha, S. *et al.* Improved annotation of the insect vector of citrus greening disease: biocuration by a diverse genomics community. *Database* **2017**, bax032 (2017).

102. Rispe, C. *et al.* The genome sequence of the grape phylloxera provides insights into the evolution, adaptation, and invasion routes of an iconic

pest. *BMC Biol.* **18**, 90 (2020).

103. Mei, Y. *et al.* InsectBase 2.0 : a comprehensive gene resource for insects. *Nucleic Acids Res.* **50**, D1040–D1045 (2022).

104. Biello, R. *et al.* A chromosome-level genome assembly of the woolly apple aphid, *Eriosoma lanigerum* Hausmann (Hemiptera: Aphididae). *Mol. Ecol. Resour.* **21**, 316–326 (2021).

105. Nicholson, S. J. *et al.* The genome of *Diuraphis noxia*, a global aphid pest of small grains. *BMC Genomics* **16**, 429 (2015).

106. Li, Y., Park, H., Smith, T. E., Moran, N. A. & Singh, N. Gene family evolution in the pea aphid based on chromosome-level genome assembly. *Mol. Biol. Evol.* **36**, 2143–2156 (2019).

107. Mathers, T. C. *et al.* Chromosome-scale genome assemblies of aphids teveal extensively rearranged autosomes and long-term conservation of the X chromosome. *Mol. Biol. Evol.* **38**, 856–875 (2021).

108. Mathers, T. C., Mugford, S. T., Hogenhout, S. A. & Tripathi, L. Genome sequence of the banana aphid, *Pentalonia nigronervosa* Coquerel (Hemiptera: aphididae) and its symbionts. *G3 Genes, Genomes, Genet.* **10**, 4315–4321 (2020).

109. Chen, W. *et al.* Genome sequence of the corn leaf aphid (Rhopalosiphum maidis Fitch). *Gigascience* **8**, 1–12 (2019).

110. Morales-Hojas, R. *et al.* Genetic structure at national and regional scale in a long-distance dispersing pest organism, the bird cherry–oat aphid

Rhopalosiphum padi. *bioRxiv* 2019.11.04.829986 (2019) doi:doi.org/10.1101/829986.

111. Wenger, J. A. *et al.* Whole genome sequence of the soybean aphid, *Aphis glycines*. *Insect Biochem. Mol. Biol.* **123**, 102917 (2017).

112. Quan, Q. *et al.* Draft genome of the cotton aphid *Aphis gossypii*. *Insect Biochem. Mol. Biol.* **105**, 25–32 (2019).

113. Li, M. *et al.* A chromosome-level genome assembly provides new insights into paternal genome elimination in the cotton mealybug *Phenacoccus solenopsis*. *Mol. Ecol. Resour.* **20**, 1733–1747 (2020).

114. Garber, A. I. *et al.* The evolution of interdependence in a four-way mealybug symbiosis. *Genome Biol. Evol.* **13**, evab123 (2021).

115. Kohli, S. *et al.* Genome and transcriptome analysis of the mealybug *Maconellicoccus hirsutus*: correlation with its unique phenotypes. *Genomics* **113**, 2483–2494 (2021).

116. Xue, J. *et al.* Genomes of the rice pest brown planthopper and its endosymbionts reveal complex complementary contributions for host adaptation. *Genome Biol.* **15**, 521 (2014).

117. Narusuye, K. *et al.* Molecular cloning of a GABA receptor subunit from *Laodelphax striatella* (Fallén) and patch clamp analysis of the homo-oligomeric receptors expressed in a *Drosophila* cell line. *Insect Mol. Biol.* **16**, 723–733 (2007).

118. Nakao, T., Hama, M., Kawahara, N. & Hirase, K. Fipronil resistance in

Sogatella furcifera: molecular cloning and functional expression of wild-type and mutant RDL GABA receptor subunits. *J. Pestic. Sci.* **37**, 37–44 (2012).

119. Tassone, E. E., Cowden, C. C. & Castle, S. J. De novo transcriptome assemblies of four xylem sap-feeding insects. *Gigascience* **6**, 1–4 (2017).

120. Ettinger, C. L. *et al.* Improved draft reference genome for the glassy-winged sharpshooter (*Homalodisca vitripennis*), a vector for Pierce's disease. *G3 Genes, Genomes, Genet.* **11**, jkab255 (2021).

121. Gupta, M. *et al.* Transcriptome analysis unravels RNAi pathways genes and putative expansion of CYP450 gene family in cotton leafhopper *Amrasca biguttula* (Ishida). *Mol. Biol. Rep.* **48**, 4383–4396 (2021).

122. Fisher, C. R., Wegrzyn, J. L. & Jockusch, E. L. Co-option of wing-patterning genes underlies the evolution of the treehopper helmet. *Nat. Ecol. Evol.* **4**, 250–260 (2020).

123. Galetto, L. *et al.* Two phytoplasmas elicit different responses in the insect vector *Euscelidius variegatus* Kirschbaum. *Infect. Immun.* **86**, e00042-18 (2018).

124. Benoit, J. B. *et al.* Unique features of a global human ectoparasite identified through sequencing of the bed bug genome. *Nat. Commun.* **7**, 10165 (2016).

125. Crava, C. M., Brütting, C. & Baldwin, I. T. Transcriptome profiling reveals differential gene expression of detoxification enzymes in a

hemimetabolous tobacco pest after feeding on jasmonate-silenced *Nicotiana attenuata* plants. *BMC Genomics* **17**, 1005 (2016).

126. Hull, J. J., Geib, S. M., Fabrick, J. A. & Brent, C. S. Sequencing and de novo assembly of the western tarnished plant bug (*Lygus hesperus*) transcriptome. *PLoS One* **8**, e55105 (2013).

127. Sparks, M. E., Shelby, K. S., Kuhar, D. & Gundersen-Rindal, D. E. Transcriptome of the invasive brown marmorated stink bug, *Halyomorpha halys* (stal) (Heteroptera: Pentatomidae). *PLoS One* **9**, e111646 (2014).

128. Sparks, M. E. *et al.* A transcriptome survey spanning life stages and sexes of the harlequin bug, *Murgantia histrionica*. *Insects* **8**, 55 (2017).

129. Genevcius, B. C. & Torres, T. T. De novo construction of a transcriptome for the stink bug crop pest *Chinavia impicticornis* during late development. *Gigabyte* **2020**, 1–7 (2020).

130. Peters, R. S. *et al.* Evolutionary history of the Hymenoptera. *Curr. Biol.* **27**, 1013–1018 (2017).

131. Vertacnik, K. L. *et al.* Ecological correlates of gene family size in a pine-feeding sawfly genome and across Hymenoptera. *bioRxiv* 2021.03.14.435331 (2021) doi:10.1101/2021.03.14.435331.

132. Oeyen, J. P. *et al.* Sawfly genomes reveal evolutionary acquisitions that fostered the mega-radiation of parasitoid and eusocial hymenoptera. *Genome Biol. Evol.* **12**, 1099–1188 (2020).

133. Robertson, H. M. *et al.* Genome sequence of the wheat stem sawfly,

Cephus cinctus, representing an early-branching lineage of the hymenoptera, illuminates evolution of hymenopteran chemoreceptors. *Genome Biol. Evol.* **10**, 2997–3011 (2018).

134. Geib, S. M., Liang, G. H., Murphy, T. D. & Sim, S. B. Whole genome sequencing of the braconid parasitoid wasp Fopius arisanus, an important biocontrol agent of pest tepritid fruit flies. *G3 Genes, Genomes, Genet.* **7**, 2407–2411 (2017).

135. Yin, C. *et al.* The genomic features of parasitism, polyembryony and immune evasion in the endoparasitic wasp *Macrocentrus cingulum*. *BMC Genomics* **19**, 420 (2018).

136. Poelchau, M. *et al.* The i5k Workspace@NAL-enabling genomic data access, visualization and curation of arthropod genomes. *Nucleic Acids Res.* **43**, D714–D719 (2015).

137. Burke, G. R., Walden, K. K. O., Whitfield, J. B., Robertson, H. M. & Strand, M. R. Whole genome sequence of the parasitoid wasp microplitis demolitor that harbors an endogenous virus mutualist. *G3 Genes, Genomes, Genet.* **8**, 2875–2880 (2018).

138. Gauthier, J. *et al.* Chromosomal scale assembly of parasitic wasp genome reveals symbiotic virus colonization. *Commun. Biol.* **4**, 104 (2021).

139. Peters, R. S. *et al.* Transcriptome sequence-based phylogeny of chalcidoid wasps (Hymenoptera: Chalcidoidea) reveals a history of rapid

radiations, convergence, and evolutionary success. *Mol. Phylogenet. Evol.* **120**, 286–296 (2018).

140. Lindsey, A. R. I. *et al.* Comparative genomics of the miniature wasp and pest control agent *Trichogramma pretiosum*. *BMC Biol.* **16**, 54 (2018).

141. Kerima, O. Z. *et al.* De novo transcriptome analysis of the egg parasitoid *Trichogramma chilonis* Ishii (Hymenoptera: Trichogrammatidae): a biological control agent. *Gene Reports* **13**, 115–129 (2018).

142. Thomas, G. W. C. *et al.* Gene content evolution in the arthropods. *Genome Biol.* **21**, 15 (2020).

143. Hoedjes, K. M., Smid, H. M., Schijlen, E. G. W. M., Vet, L. E. M. & van Vugt, J. J. F. A. Learning-induced gene expression in the heads of two Nasonia species that differ in long-term memory formation. *BMC Genomics* **16**, 162 (2015).

144. Benetta, E. D. *et al.* Genome elimination mediated by gene expression from a selfish chromosome. *Sci. Adv.* **6**, eaaz9808 (2020).

145. Yan, Z. *et al.* Insights into the venom composition and evolution of an endoparasitoid wasp by combining proteomic and transcriptomic analyses. *Sci. Rep.* **6**, 19604 (2016).

146. Xiao, J. H. *et al.* Obligate mutualism within a host drives the extreme specialization of a fig wasp genome. *Genome Biol.* **14**, R141 (2013).

147. Pauli, T. *et al.* Analysis of RNA-Seq, DNA target enrichment, and sanger nucleotide sequence data resolves deep splits in the phylogeny of cuckoo

wasps (Hymenoptera: Chrysididae). *Insect Syst. Divers.* **5**, 1 (2021).

148. Berens, A. J., Tibbetts, E. A. & Toth, A. L. Cognitive specialization for learning faces is associated with shifts in the brain transcriptome of a social wasp. *J. Exp. Biol.* **220**, 2149–2153 (2017).

149. Standage, D. S. *et al.* Genome, transcriptome and methylome sequencing of a primitively eusocial wasp reveal a greatly reduced DNA methylation system in a social insect. *Mol. Ecol.* **25**, 1769–1784 (2016).

150. Johnson, B. R. *et al.* Phylogenomics resolves evolutionary relationships among ants, bees, and wasps. *Curr. Biol.* **23**, 2058–2062 (2013).

151. Shields, E. J., Sheng, L., Weiner, A. K., Garcia, B. A. & Bonasio, R. High-quality genome assemblies reveal long non-coding RNAs expressed in ant brains. *Cell Rep.* **23**, 3078–3090 (2018).

152. Petersen, M. *et al.* Orthograph: a versatile tool for mapping coding nucleotide sequences to clusters of orthologous genes. *BMC Bioinformatics* **18**, 111 (2017).

153. Kapheim, K. M. *et al.* Genomic signatures of evolutionary transitions from solitary to group living. *Science* **348**, 1139–1144 (2015).

154. Fouks, B. *et al.* The genomic basis of evolutionary differentiation among honey bees. *Genome Res.* **31**, 1203–1215 (2021).

155. Oppenheim, S. *et al.* Whole genome sequencing and assembly of the Asian honey bee *Apis dorsata*. *Genome Biol. Evol.* **12**, 3677–3683 (2020).

156. The Honeybee Genome Sequencing Consortium. Insights into social

insects from the genome of the honeybee *Apis mellifera*. *Nature* **443**, 931–949 (2006).

157. Park, D. *et al.* Uncovering the novel characteristics of Asian honey bee, *Apis cerana*, by whole genome sequencing. *BMC Genomics* **16**, 1 (2015).

158. Sadd, B. M. *et al.* The genomes of two key bumblebee species with primitive eusocial organization. *Genome Biol.* **16**, 76 (2015).

159. Vasilikopoulos, A. *et al.* An integrative phylogenomic approach to elucidate the evolutionary history and divergence times of Neuropterida (Insecta: Holometabola). *BMC Evol. Biol.* **20**, 64 (2020).

160. McKenna, D. D. *et al.* The evolution and genomic basis of beetle diversity. *Proc. Natl. Acad. Sci. U. S. A.* **116**, 24729–24737 (2019).

161. Vasilikopoulos, A. *et al.* Phylogenomics of the superfamily Dytiscoidea (Coleoptera: Adephaga) with an evaluation of phylogenetic conflict and systematic error. *Mol. Phylogenet. Evol.* **135**, 270–285 (2019).

162. van Belleghem, S. M., Roelofs, D., van Houdt, J. & Hendrickx, F. De novo transcriptome assembly and SNP discovery in the wing polymorphic salt marsh beetle *Pogonus chalceus* (Coleoptera, Carabidae). *PLoS One* **7**, e42605 (2012).

163. i5K Consortium. The i5K initiative: advancing arthropod genomics for knowledge, human health, agriculture, and the environment. *J. Hered.* **104**, 595–600 (2013).

164. Fallon, T. R. *et al.* Firefly genomes illuminate parallel origins of

bioluminescence in beetles. *eLife* **7**, e36495 (2018).

165. Won, H. I. *et al.* De novo assembly of the burying beetle *Nicrophorus orbicollis* (Coleoptera: Silphidae) transcriptome across developmental stages with identification of key immune transcripts. *J. Genomics* **6**, 41–52 (2018).

166. Cunningham, C. B. *et al.* The genome and methylome of a beetle with complex social behavior, *Nicrophorus vespilloides* (coleoptera: Silphidae). *Genome Biol. Evol.* **7**, 3383–3396 (2015).

167. Hu, Y. H., Liu, Y., Wei, L. & Chen, H. T. De novo transcriptome sequencing of *Serangium japonicum* (Coleoptera: Coccinellidae) and application of two assembled unigenes. *G3 Genes, Genomes, Genet.* **10**, 247–254 (2020).

168. Li, H. Sen *et al.* Genomic insight into diet adaptation in the biological control agent *Cryptolaemus montrouzieri*. *BMC Genomics* **22**, 135 (2021).

169. Chen, M. *et al.* A chromosome-level assembly of the harlequin ladybird *Harmonia axyridis* as a genomic resource to study beetle and invasion biology. *Mol. Ecol. Resour.* **21**, 1318–1332 (2021).

170. Zhang, L. *et al.* Chromosome-level genome assembly of the predator *Propylea japonica* to understand its tolerance to insecticides and high temperatures. *Mol. Ecol. Resour.* **20**, 292–307 (2020).

171. Zimmer, C. T. *et al.* A de novo transcriptome of European pollen beetle populations and its analysis, with special reference to insecticide action

and resistance. *Insect Mol. Biol.* **23**, 511–526 (2014).

172. Evans, J. D. *et al.* Genome of the small hive beetle (*Aethina tumida*, Coleoptera: Nitidulidae), a worldwide parasite of social bee colonies, provides insights into detoxification and herbivory. *Gigascience* **7**, 1–16 (2018).

173. Wang, J., Hu, P., Gao, P., Tao, J. & Luo, Y. Antennal transcriptome analysis and expression profiles of olfactory genes in *Anoplophora chinensis*. *Sci. Rep.* **7**, 15470 (2017).

174. McKenna, D. D. *et al.* Genome of the Asian longhorned beetle (*Anoplophora glabripennis*), a globally significant invasive species, reveals key functional and evolutionary innovations at the beetle-plant interface. *Genome Biol.* **17**, 227 (2016).

175. Schoville, S. D. *et al.* A model species for agricultural pest genomics: the genome of the Colorado potato beetle, *Leptinotarsa decemlineata* (Coleoptera: Chrysomelidae). *Sci. Rep.* **8**, 1931 (2018).

176. Lata, D., Coates, B. S., Walden, K. K. O., Robertson, H. M. & Miller, N. J. Genome size evolution in the beetle genus Diabrotica. *bioRxiv* 2021.09.04.458993 (2021) doi:10.1101/2021.09.04.458993.

177. Bouchemousse, S., Falquet, L. & Müller-Schärer, H. Genome assembly of the ragweed leaf beetle: a step forward to better predict rapid evolution of a weed biocontrol agent to environmental novelties. *Genome Biol. Evol.* **12**, 1167–1173 (2020).

178. Sayadi, A., Immonen, E., Bayram, H. & Arnqvist, G. The de novo transcriptome and its functional annotation in the seed beetle *Callosobruchus maculatus*. *PLoS One* **11**, e0158565 (2016).

179. Nakao, T., Naoi, A., Hama, M., Kawahara, N. & Hirase, K. Concentration-dependent effects of GABA on insensitivity to fipronil in the A2′S Mutant RDL GABA receptor from fipronil-resistant *Oulema oryzae* (Coleoptera: Chrysomelidae). *J. Econ. Entomol.* **105**, 1781–1788 (2012).

180. Hu, F. *et al.* Identification and expression profiles of twenty-six glutathione S-transferase genes from rice weevil, *Sitophilus oryzae* (Coleoptera: Curculionidae). *Int. J. Biol. Macromol.* **120**, 1063–1071 (2018).

181. Noriega, D. D. *et al.* Transcriptome and gene expression analysis of three developmental stages of the coffee berry borer, *Hypothenemus hampei*. *Sci. Rep.* **9**, 12804 (2019).

182. Yuvaraj, J. K., Andersson, M. N., Zhang, D. D. & Löfstedt, C. Antennal transcriptome analysis of the chemosensory gene families from Trichoptera and basal Lepidoptera. *Front. Physiol.* **9**, 1365 (2018).

183. Kawahara, A. Y. *et al.* Phylogenomics reveals the evolutionary timing and pattern of butterflies and moths. *Proc. Natl. Acad. Sci. U. S. A.* **116**, 22657–22663 (2019).

184. Bazinet, A. L. *et al.* Phylotranscriptomics resolves ancient divergences in the Lepidoptera. *Syst. Entomol.* **42**, 305–316 (2017).

185. You, M. *et al.* A heterozygous moth genome provides insights into

herbivory and detoxification. *Nat. Genet.* **45**, 220–225 (2013).

186. Grapputo, A., Thrimawithana, A. H., Steinwender, B. & Newcomb, R. D. Differential gene expression in the evolution of sex pheromone communication in New Zealand's endemic leafroller moths of the genera Ctenopseustis and Planotortrix. *BMC Genomics* **19**, 94 (2018).

187. Sun, L.-N., Zhang, H.-J., Liu, X.-H. & Qiu, G.-S. Transcriptomic analysis of insecticide target and detoxification related genes in *Adoxophyes orana* (Lepidoptera: Tortricidae). *Acta Entomol. Sin.* **63**, 470–481 (2020).

188. Uchibori-Asano, M. *et al.* Genome-wide identification of tebufenozide resistant genes in the smaller tea tortrix, *Adoxophyes honmai* (Lepidoptera: Tortricidae). *Sci. Rep.* **9**, 4203 (2019).

189. Wei, H. *et al.* Odorant degrading carboxylesterases modulate foraging and mating behaviors of *Grapholita molesta*. *Chemosphere* **270**, 128647 (2021).

190. Cui, M., Hu, P., Wang, T., Tao, J. & Zong, S. Differential transcriptome analysis reveals genes related to cold tolerance in seabuckthorn carpenter moth, *Eogystia hippophaecolus*. *PLoS One* **12**, e0187105 (2017).

191. Challi, R., Kumar, S., Dasmahapatra, K., Jiggins, C. & Blaxter, M. Lepbase: the Lepidopteran genome database. *bioRxiv* 2016.06.06.056994 (2016) doi:10.1101/056994.

192. Nishikawa, H. *et al.* A genetic mechanism for female-limited Batesian

mimicry in Papilio butterfly. *Nat. Genet.* **47**, 405–409 (2015).
193. Li, X. *et al.* Outbred genome sequencing and CRISPR/Cas9 gene editing in butterflies. *Nat. Commun.* **6**, 8212 (2015).
194. Grishin, N. V. *et al.* Complete genome of Pieris rapae, a resilient alien, a cabbage pest, and a source of anti-cancer proteins. *F1000Research* **5**, 2631 (2016).
195. Gu, L. *et al.* Dichotomy of dosage compensation along the neo Z chromosome of the monarch butterfly. *Curr. Biol.* **29**, 4071–4077 (2019).
196. Nallu, S. *et al.* The molecular genetic basis of herbivory between butterflies and their host plants. *Nat. Ecol. Evol.* **2**, 1418–1427 (2018).
197. Antony, B. *et al.* Genes involved in sex pheromone biosynthesis of *Ephestia cautella*, an important food storage pest, are determined by transcriptome sequencing. *BMC Genomics* **16**, 532 (2015).
198. Wei, H.-S., Li, K.-B., Zhang, S., Cao, Y.-Z. & Yin, J. Identification of candidate chemosensory genes by transcriptome analysis in *Loxostege sticticalis* Linnaeus. *PLoS One* **12**, e0174036 (2017).
199. Renuka, P. *et al.* RNA-seq of rice yellow stem borer *Scirpophaga incertulas* reveals molecular insights during four larval developmental stages. *G3 Genes, Genomes, Genet.* **7**, 3031–3045 (2017).
200. Yadav, C., Smith, M. L. & Yack, J. E. Transcriptome analysis of a social caterpillar, *Drepana arcuata*: de novo assembly, functional annotation and developmental analysis. *PLoS One* **15**, e0234903 (2020).

201. Scott Chialvo, C. H. *et al.* A phylogenomic analysis of lichen-feeding tiger moths uncovers evolutionary origins of host chemical sequestration. *Mol. Phylogenet. Evol.* **121**, 23–34 (2018).

202. Gomes, F. M. *et al.* Inorganic polyphosphates are stored in spherites within the midgut of *Anticarsia gemmatalis* and play a role in copper detoxification. *J. Insect Physiol.* **58**, 211–219 (2012).

203. Dhania, N. K., Chauhan, V. K., Chaitanya, R. K. & Dutta-Gupta, A. Midgut de novo transcriptome analysis and gene expression profiling of *Achaea janata* larvae exposed with *Bacillus thuringiensis* (Bt)-based biopesticide formulation. *Comp. Biochem. Physiol. - Part D Genomics Proteomics* **30**, 81–90 (2019).

204. Fu, Y. *et al.* The genome of the Hi5 germ cell line from *Trichoplusia ni*, an agricultural pest and novel model for small RNA biology. *eLife* **7**, e31628 (2018).

205. Chen, Y.-R. *et al.* Transcriptome responses of the host *Trichoplusia ni* to infection by the baculovirus Autographa californica multiple nucleopolyhedrovirus. *J. Virol.* **88**, 13781–13797 (2014).

206. The Busseola fusca Genome Consortium. Comprehensive transcriptome of the maize stalk borer, *Busseola fusca*, from multiple tissue types, developmental stages, and parasitoid wasp exposures. *Genome Biol. Evol.* **12**, 2554–2560 (2020).

207. Oppenheim, S. J., Gould, F. & Hopper, K. R. The genetic architecture of

a complex ecological trait: host plant use in the specialist moth, Heliothis subflexa. *Evolution* **66**, 3336–3351 (2012).

208. Xiong, G. H. *et al.* High throughput profiling of the cotton bollworm Helicoverpa armigera immunotranscriptome during the fungal and bacterial infections. *BMC Genomics* **16**, 321 (2015).

209. Xu, W., Papanicolaou, A., Liu, N. Y., Dong, S. L. & Anderson, A. Chemosensory receptor genes in the oriental tobacco budworm *Helicoverpa assulta*. *Insect Mol. Biol.* **24**, 253–263 (2015).

210. Breeschoten, T., Ros, V. I. D., Schranz, M. E. & Simon, S. An influential meal: host plant dependent transcriptional variation in the beet armyworm, *Spodoptera exigua* (Lepidoptera: Noctuidae). *BMC Genomics* **20**, 845 (2019).

211. Xiao, H. *et al.* The genetic adaptations of fall armyworm *Spodoptera frugiperda* facilitated its rapid global dispersal and invasion. *Mol. Ecol. Resour.* **20**, 1050–1068 (2020).

212. Legeai, F. *et al.* An expressed sequence tag collection from the male antennae of the Noctuid moth *Spodoptera littoralis*: a resource for olfactory and pheromone detection research. *BMC Genomics* **12**, 86 (2011).

213. Gong, L. *et al.* Core RNAi machinery and three Sid-1 related genes in *Spodoptera litura* (Fabricius). *Int. J. Agric. Biol.* **17**, 937–944 (2015).

214. Li, L. T., Zhu, Y. Bin, Ma, J. F., Li, Z. Y. & Dong, Z. P. An analysis of the

Athetis lepigone transcriptome from four developmental stages. *PLoS One* **8**, e73911 (2013).

215. Ding, B. J. & Löfstedt, C. Analysis of the *Agrotis segetum* pheromone gland transcriptome in the light of sex pheromone biosynthesis. *BMC Genomics* **16**, 711 (2015).

216. Bian, H.-X. *et al.* Characterization of the adult head transcriptome and identification of migration and olfaction genes in the oriental armyworm mythimna separate. *Sci. Rep.* **7**, 2324 (2017).

217. Oppenheim, S. J., Feindt, W., DeSalle, R. & Goldstein, P. Z. De novo characterization of transcriptomes from two North American Papaipema stem-borers (Lepidoptera: Noctuidae). *PLoS One* **13**, e0191061 (2018).

218. Xiang, H. *et al.* The evolutionary road from wild moth to domestic silkworm. *Nat. Ecol. Evol.* **2**, 1268–1279 (2018).

219. Dong, Y. *et al.* Comparative transcriptome analyses on silk glands of six silkmoths imply the genetic basis of silk structure and coloration. *BMC Genomics* **16**, 203 (2015).

220. Kim, S. R. *et al.* Genome sequence of the Japanese oak silk moth, *Antheraea yamamai*: the first draft genome in the family Saturniidae. *Gigascience* **7**, 1–11 (2018).

221. Kanost, M. R. *et al.* Multifaceted biological insights from a draft genome sequence of the tobacco hornworm moth, *Manduca sexta*. *Insect Biochem. Mol. Biol.* **76**, 118–147 (2016).

222. Consortium, G. R. D. *et al.* Genomic resources notes accepted 1 February 2015 - 31 March 2015. *Mol. Ecol. Resour.* **14**, 882 (2014).

223. Mongin, E., Louis, C., Holt, R. A., Birney, E. & Collins, F. H. The *Anopheles gambiae* genome: an update. *Trends Parasitol.* **20**, 49–52 (2004).

224. Matthews, B. J. *et al.* Improved reference genome of *Aedes aegypti* informs arbovirus vector control. *Nature* **563**, 501–507 (2018).

225. Oliveira, F. *et al.* Immunity to vector saliva is compromised by short sand fly seasons in endemic regions with temperate climates. *Sci. Rep.* **10**, 7990 (2020).

226. Drosophila 12 Genomes Consortium. Evolution of genes and genomes on the *Drosophila* phylogeny. *Nature* **450**, 203–218 (2007).

227. Gloss, A. D. *et al.* Evolution of herbivory remodels a *Drosophila* genome. *bioRxiv* 2019.09.12.767160 (2019) doi:10.1101/767160.

228. Bracewell, R., Chatla, K., Nalley, M. J. & Bachtrog, D. Dynamic turnover of centromeres drives karyotype evolution in *Drosophila*. *eLife* **8**, e49002 (2019).

229. Adams, M. D. *et al.* The genome sequence of *Drosophila melanogaster*. *Science* **287**, 2185–2195 (2000).

230. Dowle, E. J. *et al.* Genome-wide variation and transcriptional changes in diverse developmental processes underlie the rapid evolution of seasonal adaptation. *Proc. Natl. Acad. Sci. U. S. A.* **117**, 23960–23969 (2020).

231. Bybee, S. M. *et al.* Phylogeny and classification of Odonata using targeted genomics. *Mol. Phylogenet. Evol.* **160**, 107115 (2021).

232. Ogden, T. H. & Whiting, M. F. Phylogeny of Ephemeroptera (mayflies) based on molecular evidence. *Mol. Phylogenet. Evol.* **37**, 625–643 (2005).

233. Song, H. *et al.* Phylogenomic analysis sheds light on the evolutionary pathways towards acoustic communication in Orthoptera. *Nat. Commun.* **11**, 4939 (2020).

234. Dietrich, C. H., Rakitov, R. A., Holmes, J. L. & Black IV, W. C. Phylogeny of the major lineages of Membracoidea (Insecta: Hemiptera: Cicadomorpha) based on ^{28}S rDNA sequences. *Mol. Phylogenet. Evol.* **18**, 293–305 (2001).

235. Kim, H. & Lee, S. A molecular phylogeny of the tribe Aphidini (Insecta: Hemiptera: Aphididae) based on the mitochondrial tRNA/COII, ^{12}S/^{16}S and the nuclear EF1α genes. *Syst. Entomol.* **33**, 711–721 (2008).

236. Huang, Y.-X., Zheng, L.-F., Bartlett, C. R. & Qin, D.-Z. Resolving phylogenetic relationships of Delphacini and Tropidocephalini (Hemiptera: Delphacidae: Delphacinae) as inferred from four genetic loci. *Sci. Rep.* **7**, 3319 (2017).

237. Munro, J. B. *et al.* A molecular phylogeny of the chalcidoidea (Hymenoptera). *PLoS One* **6**, e27023 (2011).

238. Sharanowski, B. J., Dowling, A. P. G. & Sharkey, M. J. Molecular phylogenetics of Braconidae (Hymenoptera: Ichneumonoidea), based on

multiple nuclear genes, and implications for classification. *Syst. Entomol.* **36**, 549–572 (2011).

239. Bossert, S. *et al.* Combining transcriptomes and ultraconserved elements to illuminate the phylogeny of Apidae. *Mol. Phylogenet. Evol.* **130**, 121–131 (2019).

240. Magro, A., Lecompte, E., Magné, F., Hemptinne, J. L. & Crouau-Roy, B. Phylogeny of ladybirds (Coleoptera: Coccinellidae): are the subfamilies monophyletic? *Mol. Phylogenet. Evol.* **54**, 833–848 (2010).

241. Zhang, S.-Q. *et al.* Evolutionary history of Coleoptera revealed by extensive sampling of genes and species. *Nat. Commun.* **9**, 205 (2018).

242. Shin, S. *et al.* Phylogenomic data yield new and robust insights into the phylogeny and evolution of weevils. *Mol. Biol. Evol.* **35**, 823–836 (2018).

243. Mckenna, D. D. *et al.* The beetle tree of life reveals that Coleoptera survived end-Permian mass extinction to diversify during the Cretaceous terrestrial revolution. *Syst. Entomol.* **40**, 835–880 (2015).

244. Zahiri, R. *et al.* Molecular phylogenetics of Erebidae (Lepidoptera, Noctuoidea). *Syst. Entomol.* **37**, 102–124 (2012).

245. Rubin, J. J. *et al.* The evolution of anti-bat sensory illusions in moths. *Sci. Adv.* **4**, eaar7428 (2018).

246. Regier, J. C. *et al.* A molecular phylogeny for the leaf-roller moths (Lepidoptera: Tortricidae) and its implications for classification and life history evolution. *PLoS One* **7**, e35574 (2012).

247. Kergoat, G. J. *et al.* A novel reference dated phylogeny for the genus Spodoptera Guenée (Lepidoptera: Noctuidae: Noctuinae): new insights into the evolution of a pest-rich genus. *Mol. Phylogenet. Evol.* **161**, 107161 (2021).

248. Dombroskie, J. J. & Sperling, F. A. H. Phylogeny of the tribe archipini (Lepidoptera: Tortricidae: Tortricinae) and evolutionary correlates of novel secondary sexual structures. *Zootaxa* **3729**, 1–62 (2013).

249. Léger, T., Mally, R., Neinhuis, C. & Nuss, M. Refining the phylogeny of Crambidae with complete sampling of subfamilies (Lepidoptera, Pyraloidea). *Zool. Scr.* **50**, 84–99 (2021).

250. Mitchell, A., Mitter, C. & Regier, J. C. Systematics and evolution of the cutworm moths (Lepidoptera: Noctuidae): evidence from two protein-coding nuclear genes. *Syst. Entomol.* **31**, 21–46 (2006).

251. RÖNKÄ, K., Mappes, J., Kaila, L. & Wahlberg, N. Putting Parasemia in its phylogenetic place: a molecular analysis of the subtribe Arctiina (Lepidoptera). *Syst. Entomol.* **41**, 844–853 (2016).

252. Zakharov, E. V., Caterino, M. S. & Sperling, F. A. H. Molecular phylogeny, historical biogeography, and divergence time estimates for swallowtail butterflies of the genus Papilio (Lepidoptera: Papilionidae). *Syst. Biol.* **53**, 193–215 (2004).

253. O’Grady, P. M. & DeSalle, R. Phylogeny of the genus *Drosophila*. *Genetics* **209**, 1–25 (2018).

254. Thompson, M., Steichen, J. C. & Ffrench-Constant, R. H. Conservation of cyclodiene insecticide resistance-associated mutations in insects. *Insect Mol. Biol.* **2**, 149–154 (1993).

255. Anthony, N. M., Brown, J. K., Markham, P. G. & Ffrench-Constant, R. H. Molecular analysis of cyclodiene resistance-associated mutations among populations of the sweetpotato whitefly *Bemisia tabaci*. *Pesticide Biochemistry and Physiology* vol. 51 220–228 at https://doi.org/10.1006/pest.1995.1022 (1995).

256. Zhang, Y. *et al.* Synergistic and compensatory effects of two point mutations conferring target-site resistance to fipronil in the insect GABA receptor RDL. *Sci. Rep.* **6**, 32335 (2016).

257. Nakao, T. *et al.* The A2′N mutation of the RDL γ-aminobutyric acid receptor conferring fipronil resistance in *Laodelphax striatellus* (Hemiptera: Delphacidae). *J. Econ. Entomol.* **104**, 646–652 (2011).

258. Nakao, T., Naoi, A., Kawahara, N. & Hirase, K. Mutation of the GABA receptor associated with fipronil resistance in the whitebacked planthopper, *Sogatella furcifera*. *Pestic. Biochem. Physiol.* **97**, 262–266 (2010).

259. Ffrench-Constant, R. H., Steichen, J. C. & Brun, L. O. A molecular diagnostic for endosulfan insecticide resistance in the coffee berry borer *Hypothenemus hampei* (Coleoptera: Scolytidae). *Bull. Entomol. Res.* **84**, 11–15 (1994).

260. Wang, H. *et al.* Role of a gamma-aminobutryic acid (GABA) receptor mutation in the evolution and spread of *Diabrotica virgifera virgifera* resistance to cyclodiene insecticides. *Insect Mol. Biol.* **22**, 473–484 (2013).
261. Li, A., Yang, Y., Wu, S., Li, C. & Wu, Y. Investigation of resistance mechanisms to fipronil in diamondback moth (Lepidoptera: Plutellidae). *J. Econ. Entomol.* **99**, 914–919 (2006).
262. Wang, X., Wu, S., Gao, W. & Wu, Y. Dominant inheritance of field-evolved resistance to fipronil in *Plutella xylostella* (Lepidoptera: Plutellidae). *J. Econ. Entomol.* **109**, 334–338 (2016).
263. Bass, C., Schroeder, I., Turberg, A., Field, L. M. & Williamson, M. S. Identification of the *Rdl* mutation in laboratory and field strains of the cat flea, *Ctenocephalides felis* (Siphonaptera: Pulicidae). *Pest Manag. Sci.* **60**, 1157–1162 (2004).
264. Le Goff, G., Hamon, A., Bergé, J. B. & Amichot, M. Resistance to fipronil in *Drosophila simulans*: influence of two point mutations in the RDL GABA receptor subunit. *J. Neurochem.* **92**, 1295–1305 (2005).
265. Domingues, L. N., Guerrero, F. D., Becker, M. E., Alison, M. W. & Foil, L. D. Discovery of the *Rdl* mutation in association with a cyclodiene resistant population of horn flies, *Haematobia irritans* (Diptera: Muscidae). *Vet. Parasitol.* **198**, 172–179 (2013).
266. Du, W. *et al.* Independent mutations in the Rdl locus confer dieldrin

resistance to *Anopheles gambiae* and *An. arabiensis*. *Insect Mol. Biol.* **14**, 179–183 (2005).

267. Wondji, C. S. *et al.* Identification and distribution of a GABA receptor mutation conferring dieldrin resistance in the malaria vector *Anopheles funestus* in Africa. *Insect Biochem. Mol. Biol.* **41**, 484–491 (2011).

268. Qian, W. *et al.* A survey of insecticide resistance-conferring mutations in multiple targets in *Anopheles sinensis* populations across Sichuan, China. *Parasites and Vectors* **14**, 1–10 (2021).

269. Shotkoski, F., Lee, H. -J, Zhang, H. -G, Jackson, M. B. & Ffrench-Constant, R. H. Functional expression of insecticide-resistant GABA receptors from the mosquito *Aedes aegypti*. *Insect Mol. Biol.* **3**, 283–287 (1994).

270. Tantely, M. L. *et al.* Insecticide resistance in *Culex pipiens quinquefasciatus* and *Aedes albopictus* mosquitoes from La Réunion Island. *Insect Biochem. Mol. Biol.* **40**, 317–324 (2010).

271. Hall, A. C. *et al.* Modulation of human $GABA_A$ and glycine receptor currents by menthol and related monoterpenoids. *Eur. J. Pharmacol.* **506**, 9–16 (2004).

272. Watt, E. E. *et al.* Menthol shares general anesthetic activity and sites of action on the $GABA_A$ receptor with the intravenous agent, propofol. *Eur. J. Pharmacol.* **590**, 120–126 (2008).

273. Tong, F. & Coats, J. R. Quantitative structure-activity relationships of

monoterpenoid binding activities to the housefly GABA receptor. *Pest Manag. Sci.* **68**, 1122–1129 (2012).

274. Priestley, C. M., Williamson, E. M., Wafford, K. A. & Sattelle, D. B. Thymol, a constituent of thyme essential oil, is a positive allosteric modulator of human $GABA_A$ receptors and a homo-oligomeric GABA receptor from *Drosophila melanogaster*. *Br. J. Pharmacol.* **140**, 1363–1372 (2003).

275. Nomura, K., Yoshizumi, S., Ozoe, F. & Ozoe, Y. Molecular cloning and pharmacology of Min-UNC-49B, a GABA receptor from the southern root-knot nematode *Meloidogyne incognita*. *Pest Manag. Sci.* **77**, 3763–3776 (2021).

276. Höld, K. M., Sirisoma, N. S., Sparks, S. E. & Casida, J. E. Metabolism and mode of action of cis- and trans-3-pinanones (the active ingredients of hyssop oil). *Xenobiotica* **32**, 251–265 (2002).

277. Huang, S. H. *et al.* Bilobalide, a sesquiterpene trilactone from *Ginkgo biloba*, is an antagonist at recombinant α1β2γ2L $GABA_A$ receptors. *Eur. J. Pharmacol.* **464**, 1–8 (2003).

278. Matsumura, F. & Ghiasuddin, M. Evidence for similarities between cyclodiene type insecticides and picrotoxinin in their action mechanisms. *J. Environ. Sci. Heal.* **B18**, 1–14 (1983).

279. Khom, S. *et al.* Valerenic acid potentiates and inhibits $GABA_A$ receptors: molecular mechanism and subunit specificity. *Neuropharmacology* **53**, 178–187 (2007).

280. Shinozaki, H., Ishidaa, M. & Kudoa, Y. Effects of anisatin on the GABA action in the crayfish neuromuscular junction. *Brain Res.* **222**, 401–405 (1981).

281. Kudo, Y., Oka, J.-I. & Yamada, K. Anisatin, a potent GABA antagonist, isolated from Illicium anisatum. *Neurosci. Lett.* **25**, 83–88 (1981).

282. Kuriyama, T., Schmidt, T. J., Okuyama, E. & Ozoe, Y. Structure-activity relationships of seco-prezizaane terpenoids in γ-aminobutyric acid receptors of houseflies and rats. *Bioorganic Med. Chem.* **10**, 1873–1881 (2002).

283. Hosie, A. M. *et al.* Actions of picrodendrin antagonists on dieldrin-sensitive and -resistant *Drosophila* GABA receptors. *Br. J. Pharmacol.* **119**, 1569–1576 (1996).

284. Ozoe, Y. *et al.* Picrodendrin and related terpenoid antagonists reveal structural differences between ionotropic GABA receptors of mammals and insects. *Bioorganic Med. Chem.* **6**, 481–492 (1998).

285. Bloomquist, J. R. *et al.* Mode of action of the plant-derived silphinenes on insect and mammalian $GABA_A$ receptor/chloride channel complex. *Pestic. Biochem. Physiol.* **91**, 17–23 (2008).

286. Norris, E. J. *et al.* Mode of action and toxicological effects of the sesquiterpenoid, nootkatone, in insects. *Pestic. Biochem. Physiol.* **183**, 105085 (2022).

287. Huang, S. H. *et al.* Ginkgolides, diterpene trilactones of *Ginkgo biloba*, as

antagonists at recombinant α1β2γ2L $GABA_A$ receptors. *Eur. J. Pharmacol.* **494**, 131–138 (2004).

288. Rutherford, D., Nielsen, M., Tokutomi, N. & Akaike, N. Effects of plant diterpenes on the neuronal $GABA_A$ receptor-operated chloride current. *Neuroreport* **5**, 2569–72 (1994).

289. Kessler, A. *et al.* $GABA_A$ receptor modulation by terpenoids from Sideritis extracts. *Mol. Nutr. Food Res.* **58**, 851–862 (2014).

290. Zaugg, J. *et al.* Identification of $GABA_A$ receptor modulators in *Kadsura longipedunculata* and assignment of absolute configurations by quantum-chemical ECD calculations. *Phytochemistry* **72**, 2385–2395 (2011).

291. Hossain, S. J., Aoshima, H., Koda, H. & Kiso, Y. Effects of tea components on the response of $GABA_A$ receptors expressed in *Xenopus oocytes*. *J. Agric. Food Chem.* **51**, 7568–7575 (2003).

292. Granger, R. E., Campbell, E. L. & Johnston, G. A. R. (+)- And (-)-borneol: efficacious positive modulators of GABA action at human recombinant α1β2γ2L $GABA_A$ receptors. *Biochem. Pharmacol.* **69**, 1101–1111 (2005).

293. Tong, F. & Coats, J. R. Effects of monoterpenoid insecticides on $[^3H]$-TBOB binding in house fly GABA receptor and $^{36}Cl^-$ uptake in American cockroach ventral nerve cord. *Pestic. Biochem. Physiol.* **98**, 317–324 (2010).

294. Hosseinzadeh, H. & Parvardeh, S. Anticonvulsant effects of thymoquinone, the major constituent of *Nigella sativa* seeds, in mice.

Phytomedicine **11**, 56–64 (2004).

295. Ha, J. H. *et al.* Modulation of radioligand binding to the $GABA_A$-benzodiazepine receptor complex by a new component from Cyperus rotundus. *Biol. Pharm. Bull.* **25**, 128–130 (2002).

296. Ding, J. *et al.* Curcumol from Rhizoma curcumae suppresses epileptic seizure by facilitation of $GABA_A$ receptors. *Neuropharmacology* **81**, 244–255 (2014).

297. Jäger, A. K. *et al.* Compounds from Mentha aquatica with affinity to the GABA-benzodiazepine receptor. *South African J. Bot.* **73**, 518–521 (2007).

298. Zaugg, J., Eickmeier, E., Ebrahimi, S. N., Baburin, I. & Hamburger, M. Positive $GABA_A$ receptor modulators from Acorus calamus and structural analysis of (+)-dioxosarcoguaiacol by 1D and 2D NMR and molecular modeling. *J. Nat. Prod.* **74**, 1437–1443 (2011).

299. Rueda, D. C. *et al.* Discovery of $GABA_A$ receptor modulator aristolactone in a commercial sample of the Chinese herbal drug 'Chaihu' (*Bupleurum chinense* roots) unravels adulteration by nephrotoxic aristolochia Manshuriensis roots. *Planta Med.* **78**, 207–210 (2012).

300. Singhuber, J. *et al.* $GABA_A$ receptor modulators from Chinese herbal medicines traditionally applied against insomnia and anxiety. *Phytomedicine* **19**, 334–340 (2012).

301. Lee, C. M. *et al.* Miltirone, a central benzodiazepine receptor partial

agonist from a Chinese medicinal herb *Salvia miltiorrhiza*. *Neurosci. Lett.* **127**, 237–241 (1991).

302. Kavvadias, D., Monschein, V., Sand, P., Riederer, P. & Schreier, P. Constituents of sage (*Salvia officinalis*) with in vitro affinity to human brain benzodiazepine receptor. *Planta Med.* **69**, 113–117 (2003).

303. Rutherford, D. M. *et al.* Isolation and identification from *Salvia officinalis* of two diterpenes which inhibit t-butylbicyclophosphoro[^{35}S]thionate binding to chloride channel of rat cerebrocortical membranes in vitro. *Neurosci. Lett.* **135**, 224–226 (1992).

304. Rueda, D. C. *et al.* Identification of dehydroabietc acid from *Boswellia thurifera* resin as a positive $GABA_A$ receptor modulator. *Fitoterapia* **99**, 28–34 (2014).

305. Zaugg, J. *et al.* Diterpenes from *Biota orientalis* that decrease locomotor activity in mice. *J. Nat. Prod.* **74**, 1764–1772 (2011).

306. Ortiz-Rivas, B. & Martínez-Torres, D. Combination of molecular data support the existence of three main lineages in the phylogeny of aphids (Hemiptera: Aphididae) and the basal position of the subfamily Lachninae. *Mol. Phylogenet. Evol.* **55**, 305–317 (2010).

307. Martin, N. Citrus whitefly ladybird - *Serangium maculigerum*. Interesting insects and other invertebrates. New Zealand arthropod factsheet series Number 30. http://nzacfactsheets.landcareresearch.co.nz/Index.html. Accessed 1 September 2021. (2017).

308. Tian, M., Wei, Y., Zhang, S. & Liu, T. Suitability of *Bemisia tabaci* (Hemiptera: Aleyrodidae) biotype-B and Myzus persicae (Hemiptera: Aphididae) as prey for the ladybird beetle, *Serangium japonicum* (Coleoptera: Coccinellidae). *Eur. J. Entomol.* **114**, 603–608 (2017).
