# Supplementary material for: Convergent resistance to GABA receptor neurotoxins through plant–insect coevolution
Source: Nat Ecol Evol. 2023 Jul 17;7(9):1444–56. doi: 10.1038/s41559-023-02127-4 (PMC10482695; doi:10.1038/s41559-023-02127-4)
Supplement: Supplementary file 2 — Reporting Summary [file 41559_2023_2127_MOESM2_ESM.pdf]

nature portfolio

Corresponding author(s): Jia Huang and Noah Whiteman

Last updated by author(s): June 20, 2023

Reporting Summary

Nature Portfolio wishes to improve the reproducibility of the work that we publish. This form provides structure for consistency and transparency in reporting. For further information on Nature Portfolio policies, see our Editorial Policies and the Editorial Policy Checklist.

Statistics

For all statistical analyses, confirm that the following items are present in the figure legend, table legend, main text, or Methods section.

n/a	Confirmed	
☐	☒	The exact sample size (n) for each experimental group/condition, given as a discrete number and unit of measurement
☐	☒	A statement on whether measurements were taken from distinct samples or whether the same sample was measured repeatedly
☐	☒	The statistical test(s) used AND whether they are one- or two-sided<br>*Only common tests should be described solely by name; describe more complex techniques in the Methods section.*
☒	☐	A description of all covariates tested
☐	☒	A description of any assumptions or corrections, such as tests of normality and adjustment for multiple comparisons
☐	☒	A full description of the statistical parameters including central tendency (e.g. means) or other basic estimates (e.g. regression coefficient) AND variation (e.g. standard deviation) or associated estimates of uncertainty (e.g. confidence intervals)
☐	☒	For null hypothesis testing, the test statistic (e.g. F, t, r) with confidence intervals, effect sizes, degrees of freedom and P value noted<br>*Give P values as exact values whenever suitable.*
☒	☐	For Bayesian analysis, information on the choice of priors and Markov chain Monte Carlo settings
☒	☐	For hierarchical and complex designs, identification of the appropriate level for tests and full reporting of outcomes
☒	☐	Estimates of effect sizes (e.g. Cohen's d, Pearson's r), indicating how they were calculated

Our web collection on statistics for biologists contains articles on many of the points above.

Software and code

Policy information about availability of computer code

Data collection	Rdl genes were collected from GenBank, AphidBase, InsectBase 2.0, Fireflybase, DRYAD, Lepbase, and GigaDB. The distribution of terpenoids was primarily taken from Dr. Duke's Phytochemical and Ethnobotanical Databases (http://phytochem.nal.usda.gov/ http://dx.doi.org/10.15482/USDA.ADC/1239279). The feeding data of Hemiptera were taken from The Database of British Insects and their Foodplants (Smith and Roy 2008). The feeding data of Lepidoptera were taken from the HOSTS-A Database of the World's Lepidopteran Hostplants (Robinson et al. 2021). Locomotion data were collected using Drosophila Activity Monitoring System (DAMS, Trikinetics).
Data analysis	Tree construction was performed with IQ-TREE (v1.6.12) (Minh et al. 2020). Nucleotide sequences were predicted and translated to proteins using NCBI ORFfinder (https://www.ncbi.nlm.nih.gov/orffinder/). Protein sequence alignment was performed using MUSCLE (Edgar 2004) in MEGA X (Kumar et al., 2018) or MAFFT (Katoh et al., 2002). The best-fit model was estimated using ModelFinder (Kalyaanamoorthy et al., 2017). Species tree was performed using the BUSCO pipeline and the insecta_odb10.2019-11-20 marker set (Manni et al., 2021). The richness.yule.test function in the R package ape was used to test whether ancestral shifts in Rdl copy number is associated with shifts in diversification rate given extant species richness (Paradis and Schliep 2019). Net diversification rates were estimated using the bd.ms function in the R package geiger (Magallón and Sanderson 2001). Ancestral state reconstructions were performed using PastML (Ishikawa et al., 2019). The associations between genotypes and phenotypes across the phylogeny were performed using TraitRateProp (Levy Karin et al., 2017). The sgRNAs were designed using E-CRISP (http://www.e-crisp.org/E-CRISP/) (Heigwer et al., 2014) and CHOPCHOP (https://chopchop.cbu.uib.no/) (Labun et al., 2019). Homology modeling and molecular docking were performed using Molecular Operating Environments (MOE, 2015.10). The models of RDL mutations were generated using Swiss-PdbViewer (Guex and Peitsch 1997). The pore diameters of the models were calculated using the HOLE in WinCoot (Smart et al., 1996; Emsley et al., 2010). All statistical analyses were performed using GraphPad Prism 7 (GraphPad Software).

For manuscripts utilizing custom algorithms or software that are central to the research but not yet described in published literature, software must be made available to editors and reviewers. We strongly encourage code deposition in a community repository (e.g. GitHub). See the Nature Portfolio guidelines for submitting code & software for further information.

Data

Policy information about availability of data

All manuscripts must include a data availability statement. This statement should provide the following information, where applicable:

- Accession codes, unique identifiers, or web links for publicly available datasets
- A description of any restrictions on data availability
- For clinical datasets or third party data, please ensure that the statement adheres to our policy

All source data are provided with this paper. All other raw data are available from corresponding authors on request.

Human research participants

Policy information about studies involving human research participants and Sex and Gender in Research.

Reporting on sex and gender	*Use the terms sex (biological attribute) and gender (shaped by social and cultural circumstances) carefully in order to avoid confusing both terms. Indicate if findings apply to only one sex or gender; describe whether sex and gender were considered in study design whether sex and/or gender was determined based on self-reporting or assigned and methods used. Provide in the source data disaggregated sex and gender data where this information has been collected, and consent has been obtained for sharing of individual-level data; provide overall numbers in this Reporting Summary. Please state if this information has not been collected. Report sex- and gender-based analyses where performed, justify reasons for lack of sex- and gender-based analysis.*
Population characteristics	*Describe the covariate-relevant population characteristics of the human research participants (e.g. age, genotypic information, past and current diagnosis and treatment categories). If you filled out the behavioural & social sciences study design questions and have nothing to add here, write "See above."*
Recruitment	*Describe how participants were recruited. Outline any potential self-selection bias or other biases that may be present and how these are likely to impact results.*
Ethics oversight	*Identify the organization(s) that approved the study protocol.*

Note that full information on the approval of the study protocol must also be provided in the manuscript.

Field-specific reporting

Please select the one below that is the best fit for your research. If you are not sure, read the appropriate sections before making your selection.

☒ Life sciences ☐ Behavioural & social sciences ☐ Ecological, evolutionary & environmental sciences

For a reference copy of the document with all sections, see nature.com/documents/nr-reporting-summary-flat.pdf

Life sciences study design

All studies must disclose on these points even when the disclosure is negative.

Sample size	In the bioassay and behavior experiments, sample sizes were chosen so to satisfy and exceed thresholds for statistical power. In general, sample sizes were chosen based on our recent papers (e.g. Karageorgi et al., 2019; Guo et al., 2021; Lu et al., 2022; Qiao et al., 2022)
Data exclusions	In locomotion assays, some data were excluded from the analysis because these flies died early, mostly due to dry food.
Replication	All attempts at replication were successful. Please refer to figure legend and Methods for number of replicates.
Randomization	Samples were allocated based on the corresponding genotypes.
Blinding	Investigators were not blinded to the group allocation, as each experiment required certain genotypes for experimental and control groups. Data analyzer was blinded when assessing the results. Locomotion data collection and analysis were performed using DAMS software.

Reporting for specific materials, systems and methods

We require information from authors about some types of materials, experimental systems and methods used in many studies. Here, indicate whether each material, system or method listed is relevant to your study. If you are not sure if a list item applies to your research, read the appropriate section before selecting a response.

Materials & experimental systems

n/a	Involved in the study	
☒	☐	Antibodies
☒	☐	Eukaryotic cell lines
☒	☐	Palaeontology and archaeology
☐	☒	Animals and other organisms
☒	☐	Clinical data
☒	☐	Dual use research of concern

Methods

n/a	Involved in the study	
☒	☐	ChIP-seq
☒	☐	Flow cytometry
☒	☐	MRI-based neuroimaging

Animals and other research organisms

Policy information about studies involving animals; ARRIVE guidelines recommended for reporting animal research, and Sex and Gender in Research

Laboratory animals	Wildtype and transgenic strains of Drosophila melanogaster were used in this study.
Wild animals	We did not use wild animals in this study.
Reporting on sex	We used female Drosophila melanogaster in bioassay, temperature sensitivity, and locomotion assays.
Field-collected samples	We did not use field-collected samples in this study.
Ethics oversight	We did not conduct any experiments that require ethics oversight.

Note that full information on the approval of the study protocol must also be provided in the manuscript.
